# Untargeted Metabolomics Reveals a Multi-Faceted Resistance Response to Fusarium Head Blight Mediated by the *Thinopyrum elongatum Fhb7E* Locus Transferred via Chromosome Engineering into Wheat

**DOI:** 10.3390/cells12081113

**Published:** 2023-04-08

**Authors:** Giuseppina Fanelli, Ljiljana Kuzmanović, Gloria Giovenali, Silvio Tundo, Giulia Mandalà, Sara Rinalducci, Carla Ceoloni

**Affiliations:** 1 Department of Ecological and Biological Sciences (DEB), University of Tuscia, 01100 Viterbo, Italy; giuseppina.fanelli@unitus.it (G.F.); 2 Department of Agriculture and Forest Sciences (DAFNE), University of Tuscia, 01100 Viterbo, Italy; kuzmanovic@unitus.it (L.K.); gloria.giovenali@unitus.it (G.G.); g.mandala90@gmail.com (G.M.); 3 Department of Land, Environment, Agriculture and Forestry (TESAF), University of Padova, 35020 Legnaro, Italy; silvio.tundo@unipd.it (S.T.)

**Keywords:** scab, alien introgression, breeding, untargeted metabolomics, pathway analysis, DON, GST

## Abstract

The *Thinopyrum elongatum Fhb7E* locus has been proven to confer outstanding resistance to Fusarium Head Blight (FHB) when transferred into wheat, minimizing yield loss and mycotoxin accumulation in grains. Despite their biological relevance and breeding implications, the molecular mechanisms underlying the resistant phenotype associated with *Fhb7E* have not been fully uncovered. To gain a broader understanding of processes involved in this complex plant–pathogen interaction, we analysed via untargeted metabolomics durum wheat (DW) rachises and grains upon spike inoculation with *Fusarium graminearum* (*Fg*) and water. The employment of DW near-isogenic recombinant lines carrying or lacking the *Th. elongatum* chromosome 7E region including *Fhb7E* on their 7AL arm, allowed clear-cut distinction between differentially accumulated disease-related metabolites. Besides confirming the rachis as key site of the main metabolic shift in plant response to FHB, and the upregulation of defence pathways (aromatic amino acid, phenylpropanoid, terpenoid) leading to antioxidants and lignin accumulation, novel insights were revealed. *Fhb7E* conferred constitutive and early-induced defence response, in which specific importance of polyamine biosynthesis, glutathione and vitamin B_6_ metabolisms, along with presence of multiple routes for deoxynivalenol detoxification, was highlighted. The results suggested *Fhb7E* to correspond to a compound locus, triggering a multi-faceted plant response to *Fg*, effectively limiting *Fg* growth and mycotoxin production.

## 1. Introduction

Wheat, including hexaploid bread wheat (*Triticum aestivum* L., 2n = 6x = 42) and tetraploid durum wheat (*T. durum* Desf., 2n = 4x = 28), covers the largest harvested area on earth of cereal species, ahead of maize and rice [1]. Its global grain production is currently only inferior to that of maize, but its projected consumption by 2030 is expected to greatly increase [2]. However, the prospects of satisfying the needed higher production share are strongly constrained by several factors, with climate change representing a main driver of yield volatility [2,3]. To cope with climate change, several crop species, including wheat, have undergone a shift of farming areas [4,5]. Likewise, climate change engenders the movement of pests and pathogens [6,7], leading to dramatic increases of disease burden on host crops [8]. This is the case for Fusarium diseases on wheat, particularly Fusarium head blight (FHB) or scab. FHB not only chronically causes severe epidemics in various food security hotspots where bread wheat largely predominates [8,9] but also progressively affects less conventional areas and species. One important example is that of the Mediterranean basin, where durum wheat (DW), one of the most relevant commodities, is extensively cultivated. In Mediterranean environments, alongside Fusarium crown rot, more typical of semi-arid cropping areas [9,10], the occurrence of FHB is increasingly reported [11,12,13]. FHB epidemics are also major concerns for DW cultivated in wetter and cooler areas, as in Canada and in Central-Western Europe [14]. At a global level, FHB stands out as one of the most widespread and devastating wheat diseases (e.g., [8,15]), for which breeding efforts are being given utmost priority worldwide [14,15,16,17]. However, while the use of tolerant/resistant varieties is widely acknowledged as the most robust, long-term solution (e.g., [18]) and resistance sources have been identified within cultivated and related wild Triticeae gene pools (reviewed in [16]), the goal of effective and stable FHB control remains far from being satisfactorily accomplished. This is due to the complexity of the host’s defence mechanisms, as well as the dynamics of the pathogen’s infection and epidemiology [17,19].

*Fusarium graminearum* (*Fg*), the most prominent FHB causal agent (e.g., [15,20]), is, like the majority of *Fusarium* species, a generalist pathogen [21], having a broad host range among cereals. This fact further contributes to enhancing disease incidence due to inoculum transmission through crop rotations [9,14]. Fungal spores give rise to the infection cycle upon landing on wheat heads at the flowering (anthesis) stage, with warm and humid weather conditions favouring disease establishment [17,22]. Anthers are the sites of primary infection, whose early occurrence leads to floret sterility and no seed-set. Later infected florets produce diseased kernels, mostly wilted and contaminated by trichothecene mycotoxins. These are dangerous compounds to human and animal health, the presence of which in seeds and processed food and feed represents a major additional cause of yield and quality loss and of reduced market value of harvested grains. Mycotoxins, of which the most commonly detected in FHB-affected wheat is deoxynivalenol (DON), are virulence factors that *Fusarium* pathogens produce, which, after floral invasion and hyphal spreading, reach the rachis node. Consistent evidence (e.g., [23,24] and references therein) indicates this spike section, together with the rachilla [25], as crucial for subsequent spike colonization by the fungus. It is at this level, in fact, that a main switch between a susceptible and a resistant host response occurs. In the majority of FHB-resistant wheat genotypes, a prominent event is cell wall reinforcement via lignification and callose deposition, which provides a physicochemical barrier against further fungal spread [23,26,27,28,29,30,31]. This mechanism is at the base of the commonly termed “Type II” resistance, i.e., resistance to spreading [32]. In typical Type II-resistant reactions, strong biogenesis of cell wall components is accompanied by that of enzymatic and non-enzymatic compounds able to contrast the oxidative burst caused by reactive oxygen species (ROS), representing additional signatures of the early plant defence response (see e.g., [23,31]). Antioxidant compounds include molecules that also interact with DON and other xenobiotics, leading to their detoxification (e.g., [33,34]), as in the case of the tripeptide glutathione (GSH), a key molecule for maintenance of the physiological redox state of the cell that is involved in a variety of plant stress protective pathways [35]. In bread wheat, genes for glutathione-S-transferase (GST), the enzyme that catalyses, inter alia, the formation of DON–GSH conjugates, were found to form 37 gene clusters [36], and the upregulation of *GST* genes was observed as part of the response to *Fg* inoculations by a variety of FHB-resistant genotypes [24,31,37,38]. The reduction of DON toxicity can also be brought about by DON glycosylation, catalysed by uridine-diphosphate-transferases (UGTs), which convert DON into the less toxic DON-3-d-glucoside (D3G). In wheat, either or both DON detoxification strategies turn out to be activated as part of the response to FHB ([34,39], see also ahead).

The complexity of the mechanisms and molecules involved in coping with the FHB disease corresponds to a largely multi-genic host control, with tolerant/resistant phenotypes being the result of constitutive as well as fungal-induced expression of additively acting genes at quantitative trait loci (QTL) and of their interaction with a network of background genes/QTL in addition to that with the environment. In wheat, over 600 QTL regions, distributed on all chromosomes, were found to be involved in the genetic and functional architecture of FHB resistance [15,16,40]. Although they were recently reduced to a few tens of more robust ones [41,42], the identification of candidate genes and mechanisms remains a challenging task. The search was so far focused on those genotypes and QTL regions consistently found to confer a more resistant phenotype, hence representing more relevant breeding targets. Among them, the hitherto best described and most widely deployed source of FHB genetic resistance is *Fhb1*, originally detected in the Chinese cultivar Sumai 3 and its derivatives. Since its initial mapping on the 3BS chromosome arm [43], the *Fhb1* locus was progressively narrowed down to smaller intervals in which different candidate genes for the resistant phenotype were suggested to reside [31,44,45,46,47,48]. While the molecular basis of the *Fhb1* phenotype is still disputed and elusive [49,50], most studies agree on the prevailing constitutive expression of the genes majorly involved in *Fhb1*-based resistance. This was confirmed by proteomic and, more extensively, metabolomic investigations, which indicated the reduced pathogen spread through the rachis of *Fhb1* carriers to be mainly associated with fast and strong accumulation of resistance-related metabolites belonging to shikimate and phenylpropanoid pathways capable of hindering pathogen advancement by increased host cell wall thickening, as well as antioxidant and antifungal activities [26,30,51,52,53]. The initially proposed major role in *Fhb1*-based resistance of DON-to-D3G conversion [54] remains controversial, though all studies exclude co-localization of genes for DON glycosylation with *Fhb1* [26,31,34,51,52]. Moreover, although two glutathione-S-transferases were significantly induced in an *Fhb1*-resistant line, no DON–glutathione conjugates were detected in its metabolic profile [26]. On the other hand, despite no evidence being provided of DON biotransformation products, a gene coding for a GST, in addition to others involved in cell wall reinforcement and transcriptional regulation of plant response to pathogens, was associated with *Fhb2* [27], another typical Type II FHB resistance QTL, mapped on the 6BS arm of Sumai 3 and other Asian germplasm [49,55]. 

The GSH-based detoxification route was recently invoked as a major contributor to the effective resistance conferred to wheat by wild wheat relatives belonging to the *Thinopyrum* genus [38]. This represents one of the richest sources of many useful traits for wheat improvement (reviewed in [4,56,57,58]). Resistance to Fusarium diseases is of particular value, as the currently used donor materials originate from a small pool of intraspecific germplasm, sometimes entailing yield-related drawbacks and limitations (e.g., [16,59]). Two major QTL for FHB resistance were identified in *Thinopyrum* species, one originating from the el_2_ accession of decaploid *Th. ponticum*, and the other from diploid *Th. elongatum*. The two QTL are likely orthologous, based on genetic mapping along their homoeologous group 7 long (L) arms, 7el_2_L and 7EL, respectively [60,61,62], and on partial sequence and functional homology between the *Fhb7el_2_* (named *Fhb7* by [61]) *Th. ponticum* QTL and the *Th. elongatum* locus, here referred to as *Fhb7E* [38,63,64,65]. Furthermore, *Fhb7el_2_* and *Fhb7E* confer a largely comparable phenotype when transferred into wheat, corresponding to an exceptionally high resistance to FHB spreading along the spike beyond the *Fg* inoculation point, and in addition an extremely low DON content in mature grains of recipient bread and durum wheat [60,62,66].

A GST-encoding gene was claimed to underlie the 7el_2_ *Th. ponticum Fhb7*-resistant phenotype [38]. The gene, initially identified in the genomic sequence of a *Th. elongatum* (E genome) accession (D-3458) taken as reference was proven to have a nearly identical (98%) homolog within a 245 kb 7el_2_L region, while no homolog was found in a collinear region of a 7el_1_L arm of a susceptible *Th. ponticum* accession. Expression analyses showed the *GST* gene to be constitutively expressed in all plant tissues examined and highly induced upon *Fg* spike inoculation in 7el_2_-resistant lines and in the D-3458 *Th. elongatum* accession. The presence of the *Fhb7* candidate, in contrast to its absence, was found to cause formation of a de-epoxidated DON–GSH adduct, which irreversibly impairs the toxin function [67]. Wang et al. [38] reported no homolog of *Fhb7* in the genomes of other Triticeae and plant species in general. Instead, homologs sharing up to 97% identity were found in the genomes of endophytic fungi of the *Epichloë* genus, which led to the hypothesis that the two *Thinopyrum* species had acquired the critical gene for trichothecene detoxification via horizontal transfer (HGT). A similar *GST* transcript to that of *Fhb7* was subsequently identified in the distal end of the *Th. elongatum* 7EL arm added to bread wheat cv. Chinese Spring (CS), i.e., CS-7EL [65]. This 7EL arm, originally shown to confer high FHB resistance to CS wheat [68], belongs to the Dvorak74 *Th. elongatum* accession, distinct from the D-3458 of Wang et al. [38]. Despite some structural differences between the respective *GST* sequences, a preliminary expression analysis revealed the Dvorak74-*GST* gene to be among the few 7EL-specific transcripts strongly upregulated by *Fg* infection [65]. The presence of a *GST* gene showing high homology or complete identity with the 7el_2_ *Th. ponticum Fhb7* candidate was also detected in various wheat *Th. ponticum* translocation lines and partial amphiploids [63,64]. Unexpectedly, however, the presence and expression of the *Thinopyrum* spp. *GST* gene did not match in all cases the FHB phenotype following *Fg* inoculation, with some translocation and transgenic lines for the gene in question being fully susceptible. This evidence led the authors to exclude the 7EL *GST* gene, and so its 7el_2_ homolog (*Fhb7*) was found to be the pivotal gene in conferring FHB resistance to wheat [63,64]. In fact, irrespective of their response to *Fg* infection, several other transcripts with the potential to contribute to FHB resistance were detected in the 7EL distal region [63,65]. Apart from 7EL-specific genes/products, comparative analyses at the transcriptional, biochemical and microscopic levels, focused on the rachis tissue of CS vs. CS-7EL, identified candidates for more active constitutive or induced mechanisms of resistance in the presence of 7EL involving carbon and phenylpropanoid metabolism [68,69], fortification of the plant cell wall [69,70], defence response signalling and fungal cell wall degradation [65].

The above reported studies provide evidence of some contributing factors and routes likely underlying the *Fhb7E*-linked resistant phenotype. Yet, the picture is still incomplete. Moreover, results so far produced derive mostly from analysis of either the complete 7E chromosome or its entire long arm (carrying *Fhb7E*) added to the background of the moderately susceptible CS wheat [65,69,70], circumstances that impair the possibility to focus analyses and conclusions on the target 7EL region and to end up with strongly contrasting profiles. In the present study, we used ideal genotypes for comparative analyses, i.e., DW near-isogenic recombinant lines (NIRLs) carrying or lacking a small distal segment of *Th. elongatum* 7EL (same alien source as that of CS-7EL, [65]), previously developed by chromosome engineering and proved to be highly resistant (*Fhb7E*+) or susceptible (*Fhb7E*−) to *Fg* spike point inoculation [66]. To have a comprehensive view of the impact of the presence/absence of the *Fhb7E* locus on the host (DW) metabolic makeup, both constitutive and induced by *Fg* challenge, we applied an untargeted metabolomic approach to the critical rachis tissue for FHB development and also to the mature grain of *Fhb7E* contrasting NIRLs. The results indicate several differentially represented metabolites and distinctively perturbed metabolic pathways in association with *Fhb7E*, altogether highlighting unique features of this locus besides for common elements to other major FHB resistance loci.

## 2. Materials and Methods

### 2.1. Plant Materials

**Figure 1:**
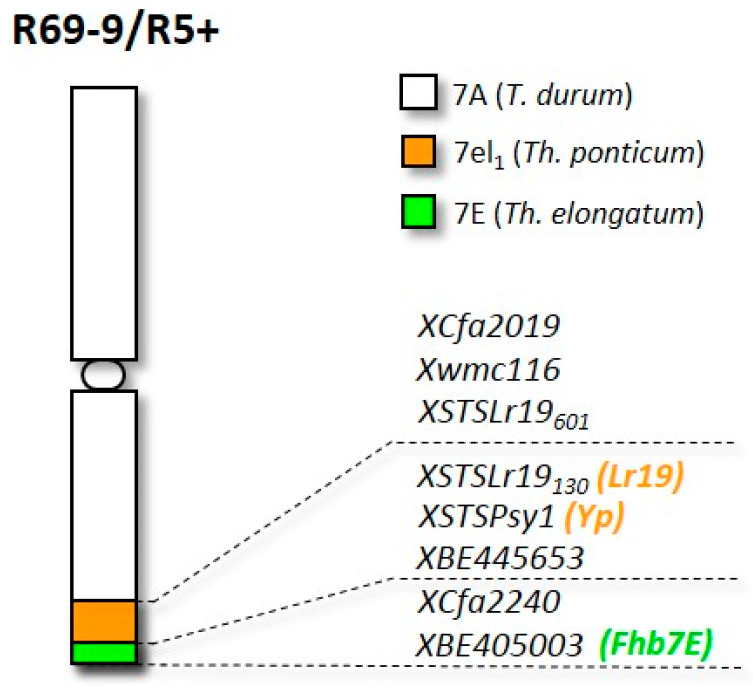
Cytogenetic map of the recombinant 7A-7el_1_/7E chromosome in the FHB-resistant R69-9/R5 HOM+ durum wheat recombinant used in the present study. Disease resistance and yellow pigment genes are indicated in the colour of the *Thinopyrum* segment of origin.

The “nested” durum wheat, namely, *Thinopyrum* ssp. homozygous recombinant line R69-9/R5+ and its control sister line R69-9/R5−, were used in the present study [66,71]. The HOM+ line has 23% of its 7AL chromosome arm pair replaced by an alien introgression composed of *Th. ponticum* (7el_1_) and *Th. elongatum* (7E) chromatin (Figure 1). In the composite alien segment, the *Th. ponticum* 7el_1_ portion is known to harbour genes for leaf rust resistance (*Lr19*) and yellow semolina colour (*Yp*) and evidently no gene contributing to FHB resistance, as from the phenotype of the primary recombinant line (R5+) carrying 7el_1_ chromatin only [66]. The FHB resistance *Fhb7E* locus was clearly associated with the most distal *Th. elongatum* 7E portion introgressed into R5+, giving rise to the R69-9/R5+ secondary recombinant [62,66]. R69-9/R5+ and R69-9/R5− plants used here, homozygous carriers (HOM+) and non-carriers (HOM−) of the composite alien segment, respectively, were selected in the same BC_3_F_2_ progeny developed in the background of durum wheat cv. Simeto. Normal transmission of the *Thinopyrum* spp. alien segment was observed (Table S1).

### 2.2. Fusarium graminearum (Fg) Inoculation

For plant infection with *Fg*, only spikes of main culms were used. Two basal florets in two opposite and alternate central spikelets were point-inoculated with 20 µL of water solution containing 1000 macroconidia of the *Fg* strain 3824 [72], freshly cultured on synthetic nutrient agar (SNA) medium, supplemented with 0.05% Tween 20. The control solution (*mock*) lacked macroconidia. The inoculation assays were conducted in controlled conditions with 16 h light/8 h dark photoperiod at 22–24 °C day and 20 °C night temperatures, when plants were at the anthesis stage (Zadoks stage 68 [73]). After inoculation, spikes were water sprayed and closed in plastic bags for 2 days to maintain high humidity. In total, 22–25 plants were inoculated per each treatment (*Fg*- or *mock*-inoculated) and genotype (HOM+ and HOM−). Disease symptoms were assessed at 7, 14 and 21 days post inoculation (dpi) by calculating the percentage number of visually diseased florets out of the total number of florets per spike. Differences in disease severity between HOM+ and HOM− genotypes were estimated by means of number of diseased florets ± SE (standard error) and by application of Student’s *t*-test at each time point. For *p*-values, 0.05, 0.01 and 0.001 significance levels were considered.

### 2.3. Tissue Sampling, Metabolite Extraction and UHPLC-MS Analysis

Rachis samples for metabolomic analysis were obtained by cutting the rachis portion associated with the four spikelets included in the inoculated spike section. Rachis tissue was immediately frozen in liquid nitrogen and stored at −80 °C until metabolite extraction. Sampling was performed at 2 and 4 dpi, as they correspond to a time range when the most active phase of the infection process occurs, accompanied by dynamic changes in the metabolome [53]. To prepare each of the three biological replicates per genotype and treatment, 2–3 rachises were combined. Rachises were grinded in liquid nitrogen and 100 mg of plant material used for metabolite extraction. Grains for the analysis were harvested at maturity from *Fg*- and *mock*-inoculated plants and milled into wholemeal flour, and 100 mg of flour was used for the extraction.

Metabolites from both rachis and grain samples were extracted in biological and technical triplicate, as previously reported [74]. Briefly, a cold (−20 °C) solution of 60% methanol/40% chloroform was added to each sample tube. The tubes were mixed for 30 min and subsequently centrifuged at 1000× *g* for 1 min at 4 °C before being transferred to −20 °C for 2–8 h. After thawing, liquid phases were recovered, and an equivalent volume of acetonitrile was added to precipitate any residual protein. Samples were then incubated at 4 °C for 20 min and centrifuged at 10,000× *g* for 10 min at 4 °C, and the collected supernatants were dried to obtain visible pellets. Finally, the dried samples were re-suspended in water containing 5% formic acid and transferred to glass autosampler vials for LC/MS analysis. Twenty microliters of extracted supernatant samples was injected into an Ultra High Performance Liquid Chromatography (UHPLC) system (Ultimate 3000, Thermo Fisher Scientific, Waltham, MA, USA) and run in positive mode: samples were loaded onto a Reprosil C18 column (2.0 mm × 150 mm, 2.5 μm—Dr. Maisch, Ammerbuch, Germany) for metabolite separation. Chromatographic separations were achieved at a column temperature of 30 °C and flow rate of 0.2 mL/min. For positive ion mode (+) MS analyses, a 0–100% linear gradient of solvent A (ddH_2_O, 0.1% formic acid) to B (acetonitrile, 0.1% formic acid) was employed over 20 min, returning to 100% A in 2 min and a 6 min post-time solvent A hold. Acetonitrile, formic acid and HPLC-grade water and standards (≥98% chemical purity) were purchased from Sigma Aldrich. The UHPLC system was coupled online with a mass spectrometer Q Exactive (Thermo Fisher Scientific, USA) scanning in full MS mode (2 μscans) at 70,000 resolution in the 67 to 1000 *m*/*z* range, with a target of 1106 ions, maximum ion injection time (IT) of 35 ms, 3.8 kV spray voltage, 40 sheath gas and 25 auxiliary gas. For DON-GSH detection, tandem mass spectrometry operated in positive ionization mode, with data dependent acquisition (DDA) mode for full-scan MS analysis. The full-scan settings were as follows: resolution 70,000; automatic gain control (AGC) target, 3 × 10^6^; maximum injection time (IT), 100 ms; and scan range, 250–3000. The remaining settings for DDA mode were as follows: resolution, 35,000; AGC target, 1 × 10^5^; isolation width, 1.7 Da; and collision energy (NCE), 30. Calibration was performed before each analysis against calibration mixes (Piercenet, Thermo Fisher, Rockford, IL, USA) to ensure sub-ppm error of the intact mass.

### 2.4. Metabolomic Data Processing and Statistical Analysis

Raw files of replicates were exported, converted into mzXML format through MassMatrix (Cleveland, OH, USA) and then processed by MAVEN 8.1 software (http://maven.princeton.edu/, accessed on 4 April 2023). Mass spectrometry chromatograms were elaborated for peak alignment, matching and comparison of parent and fragment ions, and tentative metabolite identification, within a 2 ppm mass-deviation range between observed and expected results against the imported KEGG database. Resistance-related constitutive (RRC) and resistance-related induced (RRI) metabolites with greater peak intensities in the resistant (*Fhb7E*+, HOM+) genotype in comparison to the susceptible control (*Fhb7E*−, HOM−) (fold change, FC > 1.5) were calculated as in [51]: (1) RRC = *mock* HOM+/*mock* HOM−; (2) RRI = (*Fg* HOM+/*mock* HOM+)/(*Fg* HOM−/*mock* HOM−). To further explore the metabolic differences between *Fg*- and mock-inoculated rachis samples at 2 and 4 dpi and between grain extracts of FHB-resistant and susceptible plants, multivariate statistical analyses were employed on the entire metabolomics data set using the same software. The web-based tool MetPA (Metabolomic Pathway Analysis [75]), which is incorporated into the MetaboAnalyst 5.0 platform, was used to perform pathway analyses. Data for metabolites detected in all samples were submitted into MetPA with annotation based on common chemical names. Accepted metabolites were verified manually using HMDB, KEGG and PubChem databases. The *Oryza sativa* ssp. *japonica* library was used for pathway analysis (KEGG). Global test was the selected pathway enrichment analysis method, whereas the node importance measure for topological analysis was the relative betweenness centrality. Results were graphed with GraphPad Prism 5.0 (GraphPad Software Inc., San Diego, CA, USA).

## 3. Results

### 3.1. Visual Observation of Symptoms on F. graminearum-Inoculated Spikes

**Figure 2:**
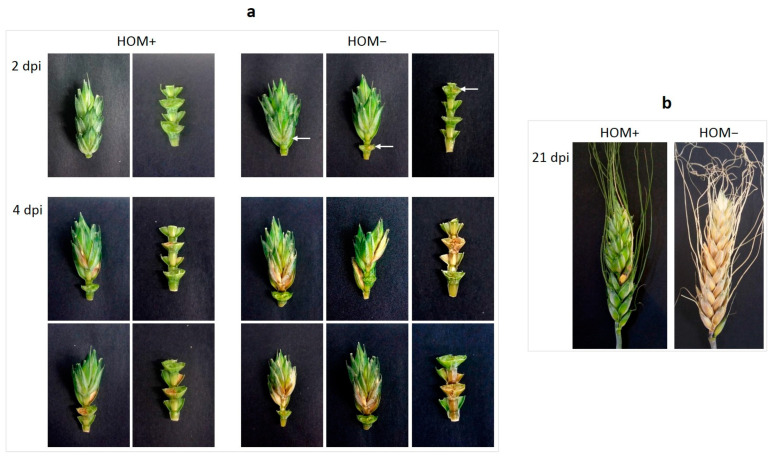
Examples of phenotypes of *Fg*-inoculated spike sections at 2 and 4 days post inoculation (dpi) (**a**) and of the whole spike at 21 dpi (**b**) for the resistant HOM+ and susceptible HOM− genotypes. White arrows indicate early browning of the inoculated floret of the susceptible genotype.

**Figure 3:**
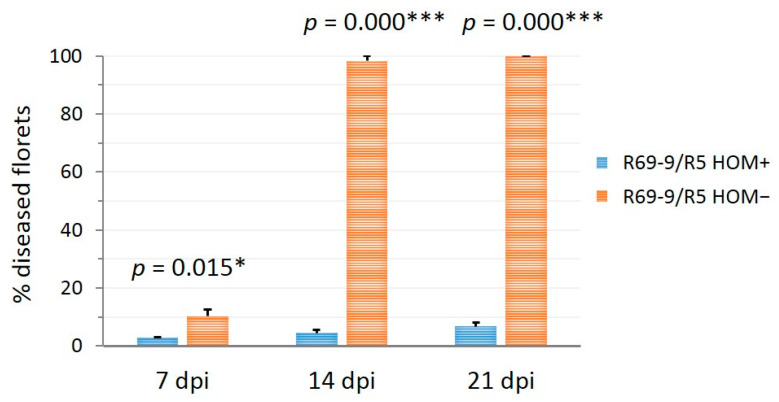
Evaluation of FHB symptom development following *Fg* inoculation in durum wheat HOM+ and HOM− lines for the *Fhb7E* locus. Data at all time points were subjected to Student’s *t*-tests, and significant probability (*p*) values are indicated by * <0.05 and *** <0.001, respectively (dpi, days post inoculation).

At 2 dpi, phenotypic differences between HOM+ and HOM− plants were hardly noticeable. *Fg*-inoculated spikelets and the associated rachis portions of the HOM+ plants did not show any visible change with respect to the *mock*-inoculated HOM+ plants (Figure 2a). On the other hand, the beginning of tissue browning could be seen at the *Fg*-inoculation site and adjacent rachis portion of HOM− plants (white arrows, Figure 2a). Differences became much more visible at 4 dpi, when disease symptoms in HOM− plants comprised in all cases floret(s) adjacent to the inoculated ones (mostly entire spikelets), and browning of the rachis extended from the *Fg*-inoculation sites toward both spike extremes (Figure 2a), clearly showing tissue colonisation by the fungus and intensive diffusion of the disease. By contrast, HOM+ plants showed browning of the inoculated florets only and did not display any symptoms on the rachis, indicating that disease spreading was stopped at the rachis nodes of the *Fg*-inoculated florets. Development of disease symptoms along the spikes until 21 dpi (Figure 2b) followed a similar dynamic to that already observed in durum wheat *Thinopyrum* spp. recombinants having the same or similar composite introgressions (all including the same *Fhb7E* locus as of HOM+) and in their HOM− sibs [66]. In the present observations, the difference in disease severity between HOM+ and HOM− lines was statistically significant at all time points, reaching 93% reduction in disease symptoms at 21 dpi in the former vs. the latter, thus confirming the exceptional efficacy of the *Fhb7E* locus from *Th. elongatum* (Figure 3). On the other hand, virtually all of the susceptible HOM− plants had 100% of diseased florets already at 14 dpi (Figure 2b).

### 3.2. Differentially Accumulated Metabolites in Rachis

**Figure 4:**
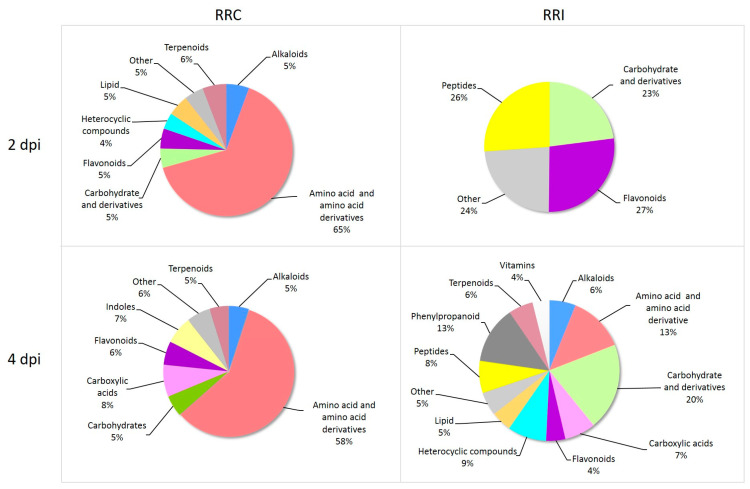
Chemical diversity of differentially accumulated metabolites at 2 and 4 days post inoculation (dpi) with water (RRC = Resistance-Related Constitutive) or *Fg* (RRI = Resistance-Related Induced), in the rachis samples of the resistant, *Fhb7E* HOM+ vs. susceptible HOM− lines.

Metabolites extracted from rachis samples at 2 and 4 dpi were analysed by liquid chromatography coupled with high-resolution accurate mass spectrometry (HRAM). Differentially expressed metabolites between HOM+ and HOM− genotypes identified in *mock*- and *Fg*-inoculated samples were indicated as resistance-related constitutive (RRC) and resistance-related induced (RRI), respectively, and the observed relative proportions are reported in Figure 4.

At 2 dpi, considering only metabolites exhibiting a FC > 1.5, a total of 61 RRC and 16 RRI differentially expressed metabolites was identified in the comparison of HOM+ vs. HOM− rachises (Figure 4, Table S2). The RRC group consisted predominantly of amino acids and related compounds (65%) and of about equally represented classes of terpenoids, flavonoids, lipids and carbohydrates and derivatives (5–6%), whereas RRI mainly included three similarly abundant classes of carbohydrates and derivatives, flavonoids, and peptides (23–27%). As for the *mock*-inoculated condition, RRC metabolites included agmatine (FC = 96.4) and citrulline (FC = 2.26) among amino acids and amino acid derivatives; gibberellin A24 (FC = 4.18) and glabric acid (FC = 2.63) within the terpenoids cluster; and taxifolin 3-O-acetate (FC = 2.34), flavonol 3-O-β-D-glucosyl-(1->2)-β-D-glucoside (FC = 2.20) and vitexin 2-O-β-D-glucoside (FC = 1.98) in the flavonoids class. On the other hand, in the *Fg*-inoculated extracts, flavonol 3-O-D-xylosylglycoside (FC = 3.08) and apigenin 7-4-dimethyl ether (FC = 1.52), metabolites of the flavonoids group, as well as glutathione (FC= 2.07) and leucyl-leucine (FC = 2.37) among peptides, were the RRI metabolites significantly more accumulated in the rachis of HOM+ than in HOM− plants.

In 4 dpi rachis extracts, the same number of classes of RRC metabolites as in the 2 dpi samples was identified, most of them having the same chemical nature and relative abundance (Figure 4, Table S3). Similar to the 2 dpi results, the major class of differentially expressed RRC metabolites in HOM+ vs. HOM− plants was that of amino acids and amino acid derivatives (58%), with terpenoids, flavonoids and carbohydrate and carbohydrate derivatives each representing 5–6%. Conversely, RRCs belonging to lipids and heterocyclic compounds were not detectable anymore, while indols and carboxylic acids emerged (7–8%). It is noteworthy that among indoles, N-hydroxyl-tryptamine (FC = 3.25) was significantly more accumulated in the HOM+ than in the HOM− genotype.

On the other hand, classes of RRI metabolites were markedly more numerous at 4 dpi (12) than at 2 dpi (4), indicating a wide spectrum of metabolic processes activated by *Fg* inoculation (Figure 4, Tables S2b and S3b), and remarkable differences in metabolic profiles of HOM+ as compared with HOM− plants were observed. In the resistant HOM+ plants, more than double the number of more abundantly accumulated RRI metabolites (143) was identified with respect to the 2 dpi time point (61, see above). This was an expected outcome of pathogen colonisation and disease development. Indeed, while the most represented chemical group of RRI metabolites remained that of carbohydrates and their derivatives (20%), flavonoids and peptides decreased by about 7 and 3 times, respectively, with respect to the 2 dpi time point (Figure 4, right panels). Moreover, phenylpropanoids (13%), terpenoids (6%), lipids (5%) and vitamins (4%), were newly detected compound classes. They are known to include metabolites either involved in cell wall reinforcement or acting as antifungal/antimicrobial compounds against pathogen attack, with crucial roles in FHB resistance in wheat (e.g., [27]). Specific RRI metabolites of each chemical class that were markedly more abundant in the resistant HOM+ vs. the HOM− genotype are described below (see also Table S3). Among the classes containing RRIs with the highest FC increase were those of phenylpropanoids (e.g., N-caffeoylputrescine (FC = 35.94), 5-o-feruloyquinic acid (FC = 7.41), 4-prenylresveratrol (FC = 6.37), sinapyl alcohol (FC = 3.13)) and carboxylic acids (shikimate (FC = 58.45), caffeoquinone (FC = 6.23), chorismate (FC = 1.83). The flavonoids class, in turn, was one of those with more numerous RRI, comprising methylquercetin (FC = 4.21), 6-methoxy taxifolin (FC = 2.83), quercetin 3-O-(6-O-malonyl-β-D-glucoside) (FC = 2.45), anthocyanidin (FC = 2.40) and lupinosoflavone G (FC = 1.87). Among the ten mono-, di-, tri- and sesqui-terpenoids and their glucose conjugates, brusatol (FC = 4.44), gibberellin A12 aldehyde (FC = 4.18) and gibberellin A5 (FC = 3.99) were the most noteworthy. Chitobiose (FC = 2.69), among the carbohydrates and derivatives; glutathione (FC = 3.39; upregulated also at 2 dpi) and glutathione disulfide (FC = 2.28), belonging to the peptides class; and 1-oleoyl-glycerophosphocholine (FC = 7.25) and cucurbic acid (FC = 2.01), of the lipid class were also significantly more accumulated in HOM+ than in HOM−. Finally, the higher abundance of six vitamins, i.e., thiamine aldehyde (FC = 2.98; vitamin B_1_), menaquinone (FC = 2.86; vitamin K_2_), pantothenol (FC = 1.60; vitamin B_5_) and vitamin B_6_ (VB6) vitamers (pyridoxine (FC = 2.26), pyridoxal phosphate (FC = 2.02), and pyridoxine phosphate (FC = 1.53)), were specifically detected in the resistant HOM+ and not in susceptible HOM− rachises.

#### Pathway Analysis

**Figure 5:**
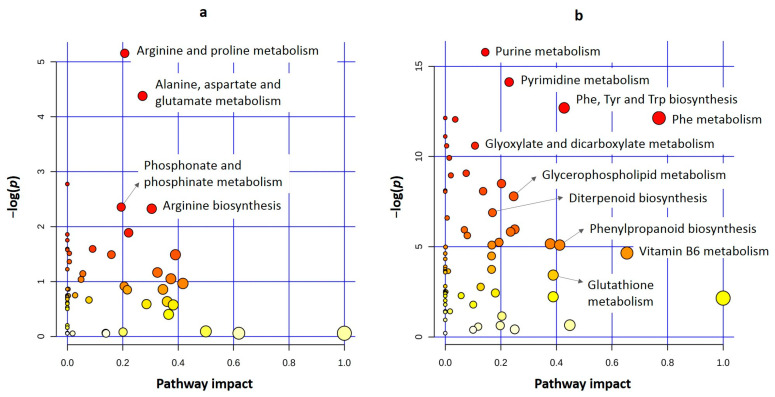
Metabolomics Pathway Analysis (MetPA) of rachis tissue from the resistant *Fhb7E* (HOM+) and susceptible (HOM−) lines at 4 dpi after water (*mock*) or *Fg* treatment: (**a**) *mock*-HOM+ vs. *mock*-HOM−, and (**b**) *Fg*-HOM+ vs. *Fg*-HOM− comparisons. All matched pathways are displayed as circles. The colour of each circle is based on *p*-values (darker colours indicate more significant changes of metabolites in the corresponding pathway), whereas the circle size corresponds to the pathway impact score. The most impacted pathways (high statistical significance scores) are annotated by their full name (Phe, phenylalanine; Tyr, tyrosine; Trp, tryptophan).

To better understand the functional roles of the identified specific RRC and RRI metabolites in the response to *Fg* infection, a detailed analysis of altered metabolic pathways and networks was carried out on 4 dpi extracts. The web-based tool MetPA was used to analyse the following comparisons: HOM+ *Fg*-inoculated vs. HOM+ *mock* (Figure S1a), HOM− *Fg*-inoculated vs. HOM− *mock* (Figure S1b), HOM+ *mock* vs. HOM− *mock* (Figure 5a) and HOM+ *Fg*-inoculated vs. HOM− *Fg*-inoculated (Figure 5b).

**Figure 6:**
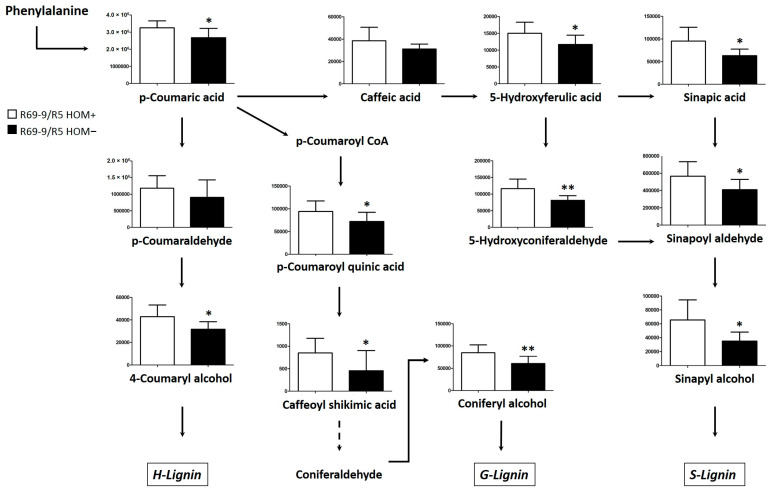
Significant changes in accumulation of intermediates of the phenylpropanoid biosynthetic pathway in the resistant *Fhb7E* (HOM+) vs. susceptible (HOM−) lines at 4 dpi (*Fg*). Values on y-axes always indicate *Peak intensity* of a given metabolite, while bars on histograms represent standard deviations of means. Comparison for each metabolite between HOM+ and HOM− genotype was analysed using Student’s *t*-test; * and ** indicate *p*-values at <0.05 and <0.01 levels of significance, respectively. Solid arrows between metabolites indicate a direct transforming relationship, while the dotted arrow indicates an indirect process (more than one step).

As for the constitutive pathways, i.e., those independent of *Fg* inoculation, the HOM+ *mock* vs. HOM− *mock* comparison (Figure 5a) revealed significant alterations (FDR < 0.05; pathway impact values > 0.1) in two amino acid pathways: one of “Arginine and proline metabolism” (downregulated in HOM+ vs. HOM−), and the other of “Alanine, aspartate and glutamate metabolism” (upregulated in HOM+ vs. HOM−). This was in line with the results of differential metabolite classes (Figure 4). On the other hand, pathway enrichment and topology analysis of HOM+ vs. HOM− *Fg*-inoculated samples identified 20 metabolic routes that were significantly perturbed under *Fg* inoculation (Figure 5b) and indicated metabolites whose higher accumulation was induced in the resistant genotype. According to the categorization made by FC analysis, evidence from MetPA showed main changes between HOM+ and HOM− *Fg*-inoculated samples at the level of metabolites belonging to aromatic amino acid metabolism (Phe, Tyr, Trp), phenylpropanoid and diterpenoid biosynthesis, glutathione metabolism and VB6 metabolism. In particular, within the induced phenylpropanoid pathway, marker metabolites such as p-coumaric acid, coumaroyl quinic acid and sinapic acid were observed in significantly higher quantity in samples of the resistant HOM+ plants, indicating that lignin biosynthesis is activated under *Fg* infection (Figure 6).

**Figure 7:**
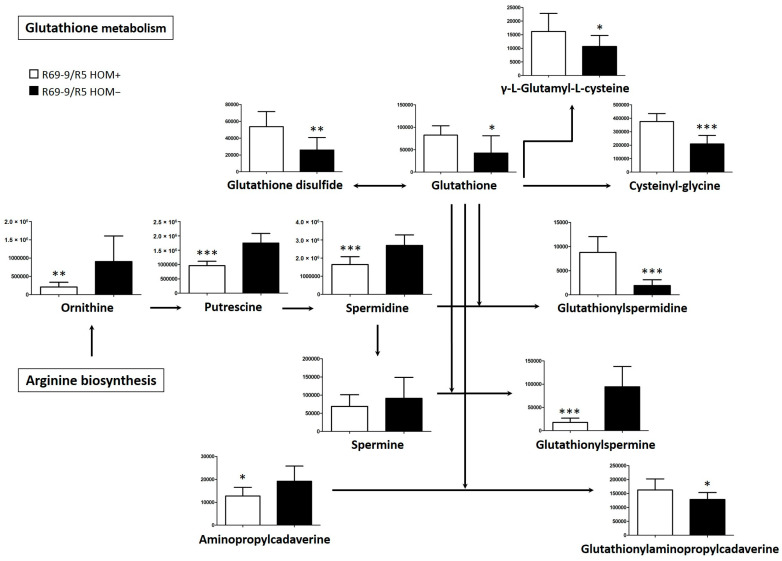
Significant changes in accumulation of intermediates of glutathione metabolism in rachis tissue of the resistant *Fhb7E* (HOM+) and susceptible (HOM−) lines at 4 dpi (*Fg*) and their connection with the polyamine biosynthesis pathway. Values on y-axes always indicate *Peak intensity* of a given metabolite, while bars on histograms represent standard deviations of means. Comparison for each metabolite between the HOM+ and HOM− genotypes was analysed using Student’s *t*-test; *, ** and *** indicate *p*-values at <0.05, <0.01, and <0.001 levels of significance, respectively. Arrows indicate a direct process between molecules.

In fact, this was paralleled by the significantly higher accumulation of downstream metabolites, i.e., coniferaldehyde and three hydroxycinnamyl alcohols or monolignols (coniferyl alcohol, sinapyl alcohol, and p-coumaryl alcohol), which are the major building blocks of the three lignin types, i.e., G, S and H (Figure 6). The induced increase in the amount of glutathione and some of its derivatives in the resistant HOM+ plants at both 2 dpi and 4 dpi (see above) was confirmed also by the observed significant perturbation of its metabolism/pathway with respect to the susceptible HOM− control under *Fg* infection (Figure 5 and Figure S1). In particular, in addition to an increase of glutathione and its precursor gamma-glutamylcysteine (γ-EC), which highlights active glutathione synthesis (Figure 7), a marked upregulation in abundance of two metabolites derived from polyamines, namely, glutathionyl-spermidine and glutathionyl-aminopropylcadaverine, both linked to glutathione metabolism, was registered (Figure 7). 

**Figure 8:**
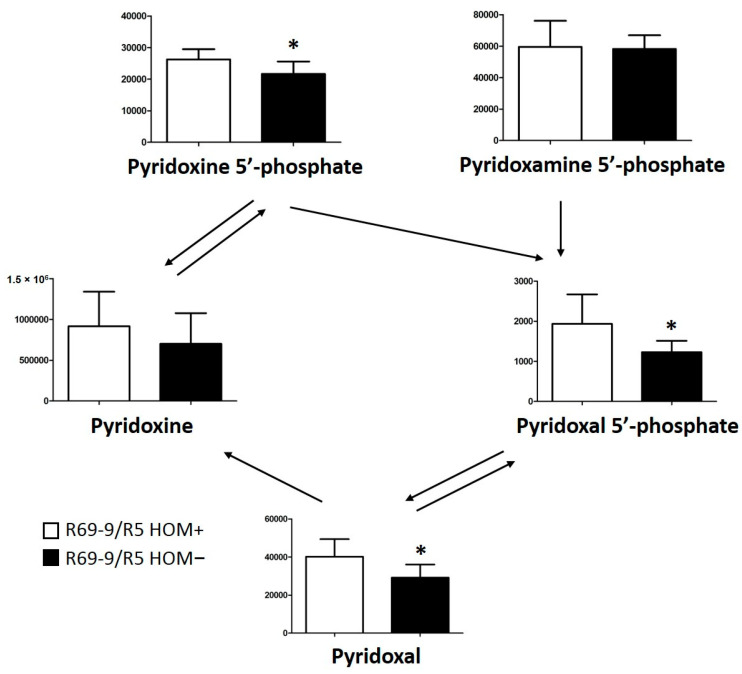
Significant changes in accumulation of intermediates of vitamin B_6_ in rachis tissue of the resistant *Fhb7E* (HOM+) and susceptible (HOM−) lines at 4 dpi (*Fg*). Values on y-axes always indicate *Peak intensity* of a given metabolite, while bars on histograms represent standard deviations of means. For each metabolite, comparison between the HOM+ and HOM− genotypes was analysed using Student’s *t*-test; * indicates *p*-value at the <0.05 level of significance. Arrows between metabolites indicate a direct transforming relationship. Double arrows denote a reversible reaction.

An additional metabolic feature that turned out to be characteristic of the resistant HOM+ plants’ response to *Fg* involved the VB6 metabolism. Accumulation of some VB6 vitamers, i.e., pyridoxine (PN) and its derivatives pyridoxal (PL) and pyridoxal 5′-phosphate (PLP), was significantly induced in HOM+ plants (Figure 8). The specific increase in VB6 accumulation in HOM+ plants under *Fg* infection was also confirmed by the pathway analysis in the *Fg*-HOM+ vs. *mock*-HOM+ comparison (Figure S1a). This evidence, which was not often observed in plant–pathogen interactions in crop species (e.g., [76]), suggests an association of the VB6 metabolism boost with *Fhb7E*-linked resistance to *Fg*.

### 3.3. Grain Tissue Metabolite Profiling

**Figure 9:**
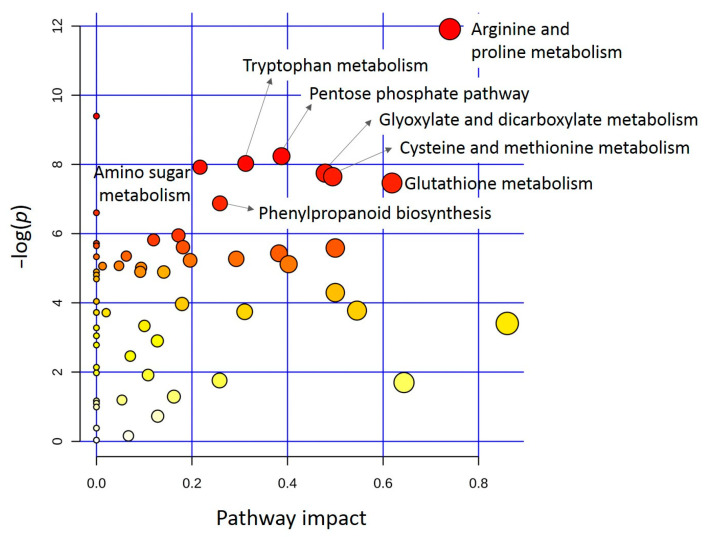
Metabolomics Pathway Analysis (MetPA) of grain tissue from the resistant *Fhb7E* (HOM+) vs. susceptible (HOM−) lines at maturity, after inoculation with *Fg*. All the matched pathways are displayed as circles. The colour of each circle is based on *p*-values (darker colours indicate more significant changes of metabolites in the corresponding pathway), whereas the size of the circle corresponds to the pathway impact score. The most impacted pathways, having high statistical significance scores, are annotated by their full name.

**Figure 10:**
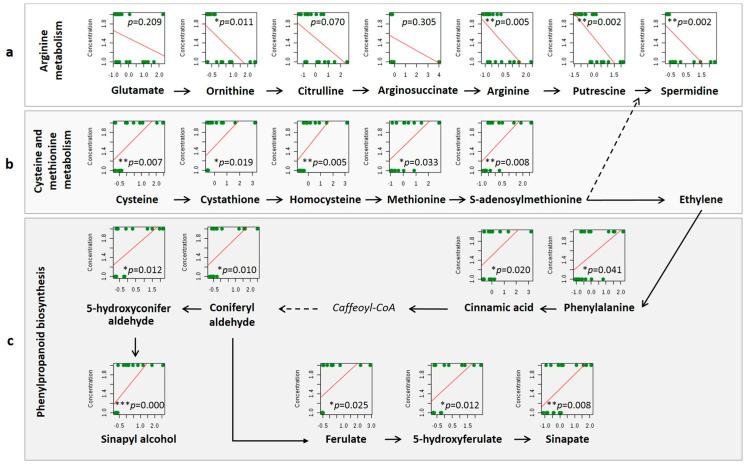
Significant changes in accumulation of intermediates of “Arginine metabolism” (**a**), “Cysteine/methionine metabolism” (**b**) and the “Phenylpropanoid biosynthetic pathway” (**c**) in *Fg*-inoculated grain tissues of the resistant *Fhb7E* (HOM+) vs. susceptible (HOM−) lines. The distribution of the compound concentration, as from Metaboanalyst 5.0 software (red lines), was analysed using Student’s *t*-test. *, ** and *** indicate *p*-values at <0.05, <0.01 and <0.001 levels of significance, respectively. Solid arrows between metabolites indicate a direct transforming relationship, while dotted arrows indicate an indirect process (more than one step).

Similarly to the analysis of rachis tissue, differentially modulated metabolites were mapped into metabolic pathways for the grain tissue through the MetPA bioinformatic pathway enrichment tool. At least six metabolic pathways with high significance and high pathway impact were identified in the *Fg*-inoculated HOM+ compared with the HOM− samples (Figure 9). These included “Phenylpropanoid biosynthesis”, “Glyoxylate and dicarboxylate metabolism”, “Glutathione metabolism” and “Tryptophan metabolism”, which were also significantly different between HOM+ and HOM− rachis tissues (see above in previous sections), as well as “Metabolisms of arginine and proline” and of “Cysteine and methionine”, the latter being specific to the grain tissue (Figure 9 and Figure 10a,b). As for “Phenylpropanoid biosynthesis” (Figure 10c), a similar trend was observed in grain as in the rachis tissue, with a generally higher accumulation of metabolites downstream of phenylalanine biosynthesis in resistant HOM+ vs. susceptible HOM− plants. However, while different paths leading to the accumulation of at least three types of lignin were upregulated in rachis (Figure 6), only the one resulting in hydroxycinnamic acids accumulation was highlighted in grains, comprising derivatives of cinnamic acid, i.e., ferulate and sinapate (Figure 10c).

The metabolic pathway with the highest impact in grains was that of “Arginine and proline metabolism” (Figure 9), which was significantly downregulated in HOM+ as compared with HOM− plants (Figure 10a), similarly to what observed in the *mock*-treated rachis tissue (Figure 5a). Compounds such as arginine, putrescine and spermidine were less abundant in grains of the resistant genotype. This result is in line also with the reduced accumulation of the same compounds in the HOM+ but not in HOM− *Fg*-inoculated rachis (Figure 7 and Figure S1), indicating these metabolites as markers of the early FHB resistance response. The fact that “Arginine and proline metabolism” was also one of the few significant and differential metabolisms constitutively expressed by the resistant genotype indicates the FHB resistance component to be inherently present in the carrier line of the *Fhb7E* locus. On the other hand, “Cysteine and methionine metabolism” and its components (cysteine, cystathione, homocysteine and methionine) were found to be upregulated in grains of resistant HOM+ vs. susceptible HOM− plants, and their increase was correlated with the downstream increase of phenylpropanoid biosynthesis (Figure 10).

### 3.4. DON Detoxification Mechanisms in Rachis and Grain Tissue

**Figure 11:**
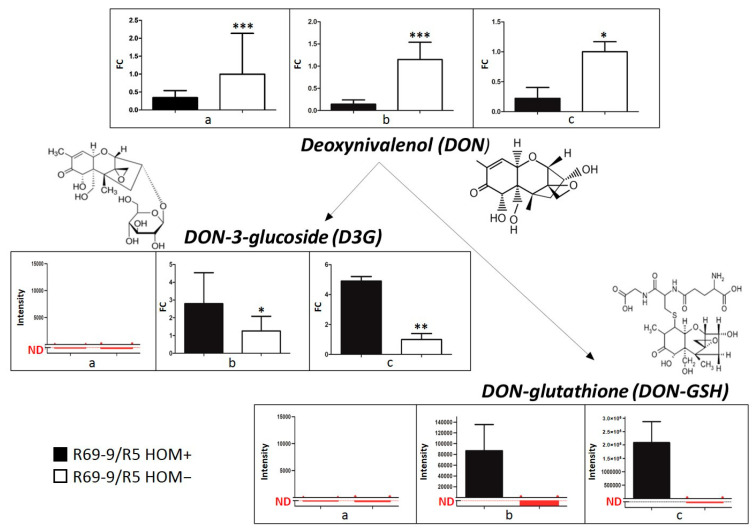
Relative quantities (FC, intensity) of DON and its D3G and DON–GSH conjugates in HOM+ and HOM− *Fg*-inoculated rachises at 2 dpi (**a**) and 4 dpi (**b**), and in mature grains (**c**). Red dotted lines indicate no detection (ND), i.e., the metabolite is not present or present in minimal amounts, not distinguishable from the instrument’s background noise. At each time point × tissue combination, the data of HOM+ vs. HOM− *Fg*-inoculated samples were analysed using Student’s *t*-test at *p* < 0.05 *, 0.01 ** and 0.001 *** significance levels. Arrows indicate possible DON biotransformations.

The presence of DON and of its glucoside- and glutathione-conjugated derivatives (D3G and DON–GSH, respectively), both representing detoxified products, was investigated in HOM+ and HOM− infected rachises and grains. At 2 dpi (Figure 11a), DON was detected in rachises of both genotypes, yet showing an average 60% decrease in HOM+ compared with HOM− samples. In the same samples, neither D3G nor DON–GSH was detected (Figure 11). By contrast, at 4 dpi (Figure 11b), while DON relative abundance further decreased in HOM+ vs. HOM− infected samples (by approximately 80%), both D3G and DON–GSH were present in HOM+ *Fg*-inoculated samples, the latter being exclusively detected in the resistant HOM+ genotype. These results indicated the existence of at least two different pathways for DON detoxification controlled by the *Fhb7E* locus. To substantiate the binding of the GSH group to the DON C13 carbon, tandem mass spectrometry experiments were performed on 4 dpi samples of infected HOM+ and HOM− rachises. The results showed the clear presence of DON–GSH-extracted ion chromatograms (EICs) in the resistant HOM+ genotype and their complete absence in susceptible HOM− (Figure S2), thus unequivocally supporting the DON–GSH enzymatic origin of the detected *Fhb7E*-specific adduct [38]. The relative accumulation of DON, D3G and DON–GSH was determined also in grains harvested from HOM+ and HOM− *Fg*-inoculated plants (Figure 11c). The results clearly showed DON content to be significantly lower (70%) in HOM+ plants than in HOM− sibs, while the reverse was true for D3G (5-fold higher in HOM+).

## 4. Discussion

**Figure 12:**
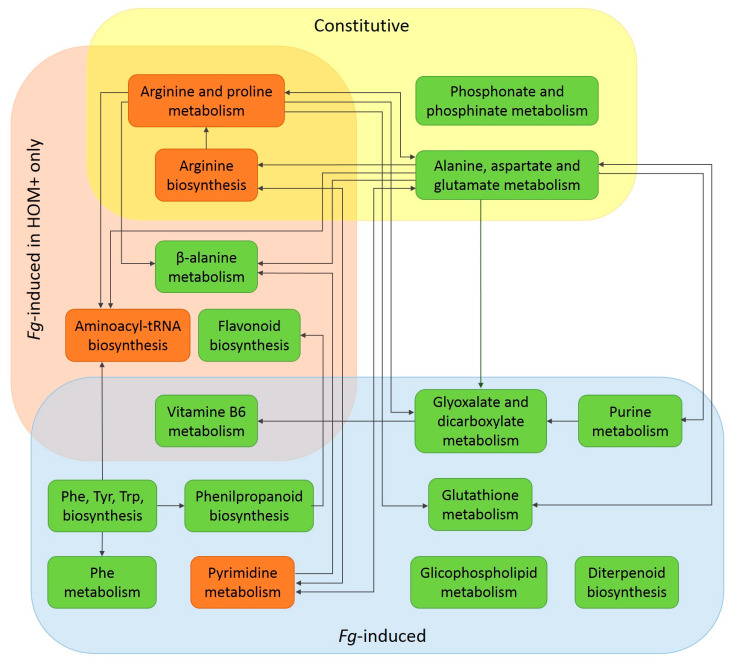
Differentially regulated pathways in rachis of the FHB-resistant HOM+ vs. susceptible HOM− lines at 4 dpi. *Red* and *green* squares indicate downregulated and upregulated pathways, respectively, in the HOM+ genotype, as from the comparisons performed and reported in Figure 5 and Figure S1a. Arrows indicate putative crosstalk between pathways in accordance with the KEGG database (Phe, phenylalanine; Tyr, tyrosine; Trp, tryptophan).

To shed light on the mechanisms of action underlying the potent resistance response against FHB associated with the introgression of the *Th. elongatum* FHB resistance locus *Fhb7E* into durum wheat, we analysed the metabolic profiles of rachis and grain tissues of a pair of contrasting near-isogenic lines, one carrying (HOM+) and the other lacking (HOM−, the *Fhb7E* locus. The untargeted metabolomics-based comparison allowed for the identification of constitutive and induced metabolites and pathways differentially expressed in the two genotypic alternatives and hence linked to the alien introgression. As a whole, a clear difference between the resistant and susceptible genotypes was observed in both tissues explored, particularly following the exposure to the pathogen. The observed differences affected various aspects of plant response, including promptness, magnitude and complexity. These variations could be better perceived at the rachis level, where the HOM+ genotype was found to appreciably respond to *Fg*-inoculation already at 2 dpi and to activate a significantly more complex matrix of metabolic pathways compared with the susceptible HOM− line at 4 dpi (Figure 12). As is typical for the wheat FHB resistance response controlled by other QTL such as *Fhb1* or *Fhb2* (see Introduction), it was at the rachis level that pathogen diffusion was with high efficacy hindered in the resistant *Fhb7E* HOM+ recombinant as a consequence of activation of a strong antioxidant response, modifications of the secondary cell-wall structure and trichothecene detoxification mechanisms. Our research confirmed the significance of GST-mediated DON detoxification, apparently associated with a fungal-to-plant HGT event ([38] and Introduction), as one of the most prominently activated in the metabolic profile of the *Fhb7E*-resistant HOM+ genotype, but it also revealed other important roles of glutathione as well as of other molecules in the *Fhb7E*-mediated defence response. On the other hand, while Wang et al. [38] analysed *Fhb7* expression in bread wheat *Th. ponticum* recombinants containing introgressions from the *Th. ponticum* el_2_ genome, we employed a durum wheat genotype incorporating the original *Th. elongatum* 7E allele (*Fhb7E*) derived from the Dvorak74 accession (see also [65]). The 7el_2_ and 7E homoeoloci were previously mapped by our group at corresponding physical locations on the distal end of the respective chromosome arms in both durum and bread wheat recombinants [60,62,66]. The largely similar phenotypes determined by the two *Fhb7* loci (70–85% and >90% reduction of disease severity associated with the *Fhb7el_2_* and *Fhb7E*, respectively) further supported their orthologous nature, as confirmed by other authors (e.g., [38,61,64]). Therefore, our results essentially represent the first characterization of the metabolic effects produced by the *Fhb7E* locus from the original diploid *Th. elongatum* donor, both at the initial entry point of the *Fg* pathogen (rachis), as well as at the level of the plant sink organ (grain).

### 4.1. Constitutive Defence Potential of Fhb7E-Carrier Lines

Pathway analysis of rachis tissues revealed that negative regulation of polyamine biosynthesis (“Arginine biosynthesis and metabolism”) and positive regulation of amino acid and phospholipid synthesis (“Alanine, aspartate and glutamate” and “Phosphonate and phosphinate” metabolisms) under *mock*-treatment (Figure 12) were apparently critical for the HOM+ recombinant defence potential, as further highlighted under *Fg*-infection. In fact, the inherently increased activity of pathways providing precursors for synthesis of antioxidant, signalling and cell membrane molecules, e.g., “Glyoxalate and dicarboxylate/Glutathione”, “β-alanine” and “Glycophospholipid” metabolisms, correlated with the upregulated pathways under *Fg* challenge. Intrinsic levels of glutamate were reported to be associated with the positive modulation of tomato resistance to *F. oxysporum* var. *lycopersicum* [77] and, in general, with an early response and adaptation of plants to pathogen stress by inducing the expression of resistance genes and the synthesis of antioxidant molecules such as glutathione (reviewed in [78]). Moreover, glutamate metabolism was identified as one of the most altered ones under *Fg* infection in bread wheat germplasm containing *Fhb1* and/or *Qfhs.ifa-5A* FHB resistance loci [79]. Specifically, the presence of *Qfhs.ifa-5A* seemed to confer better endurance under *Fg* attack by providing greater influx of amino acids as substrates for secondary metabolites, which is in line with our results. 

Metabolite composition analysis (Figure 1) showed additional specific alterations in the HOM+ vs. HOM− comparison at both time points analysed, with more numerous differential metabolites displayed by the resistant genotype at 4 than at 2 dpi. The HOM+ genotype had constitutively higher accumulation of protective metabolites with a well-established role in plant–pathogen interactions (RRC), such as flavonoids, terpenoids and alkaloids, and amino acid-related compounds (Figure 1). This is indicative of a better potential for defence and maintenance of vital biosynthetic mechanisms, as sustained by the observed significant accumulation of specific RRC metabolites, such as agmatine (FC = 96.3) and the plant hormone gibberellin A24 (FC = 4.18) at 2 dpi. Gibberellins are known to increase resistance to necrotrophic pathogens in wheat and barley by the fine tuning of ROS and inducing changes in the cell wall [80], while agmatine is a precursor of polyamine biosynthesis, a key signalling factor in plant–pathogen interactions. The roles of agmatine, better understood in animal than in plant cells, include inhibition of cell proliferation, NO-synthase and polyamine transport into cells [81]. In plants, agmatine is believed to act as a strong inducer of trichothecene biosynthesis under *Fg* infection [82,83], which may seem in contrast with its remarkably high accumulation observed in our resistant HOM+ plants (RRC, 2 dpi). On the other hand, a recent reconsideration of agmatine roles [84] demonstrated trichothecene biosynthesis by *F. graminearum* not to be stimulated by simple agmatine presence, but rather by a continuous acidification arising from agmatine catabolism. Therefore, as agmatine here was exclusively found as a constitutively accumulated metabolite in the resistant HOM+ (*mock*), it is probable that under *Fg* infection, agmatine is prevented from being metabolised to putrescine, as it commonly occurs. This is in line with the significantly lower concentration of polyamines (putrescine, spermine, spermidine) observed at 4 dpi in HOM+ vs. HOM− plants under *Fg* infection (Figure 7), with significantly downregulated “Arginine and proline metabolism” (Figure 5a) and inhibition of DON biosynthesis by the fungus (Figure 11). Agmatine accumulation was previously found to increase in bread wheat resistant cv. Sumai 3 under *Fg* infection [53], which indicates the importance of the spermine route also in *Fhb1*-associated resistance. Moreover, continuous agmatine production was also reported in *Arabidopsis* plants partially resistant to *Plasmodiophora brassicae* infection [85] and disease symptom development associated with differences in arginine metabolism and agmatine production, in line with the present observation in durum wheat upon *Fg* infection.

### 4.2. Induced Response of Fhb7E-Carrier Lines to Fg Infection

#### 4.2.1. *Fg*-Induced Metabolic Changes in Rachis

When challenged by *Fg*, the resistant response of the *Fhb7E* HOM+ genotype expressed at the rachis tissue was characterised by the upregulation of most of the pathways involved in the synthesis of protective molecules (Figure 12): lignins for secondary cell wall reinforcement (“Phenilpropanoid biosynthesis”, “Phenylalanine metabolism”), glycophospholipids for membrane integrity (“Glycophospholipid metabolisms”), glutathione (“Glutathione metabolism”) and vitamins (“Vitamin B_6_ metabolism”) for ROS neutralisation and xenobiotics detoxification, in addition to alkaloids and gibberellins (“Diterpenoid” and “Phe, Tyr, Trp” biosynthesis) for antimicrobial activity. The highly significant response of the HOM+ genotype was already evident at 2 dpi, when strong antioxidant activity and a higher accumulation of peptides and flavonoids were detected (Figure 1, RRC). Among such compounds were glutathione (FC = 2.05) and apigenin (FC = 1.74), both known to increase wheat resistance to FHB and to decrease mycotoxin production [34,86,87]. Glutathione, in particular, is well known to be involved in a myriad of metabolic processes, including detoxification of xenobiotics and ROS elimination via redox reactions [35]. Similarly, flavonoids act as quenchers of ROS, induce a hypersensitive reaction at early infection stages [88] and protect plant cell wall integrity by inhibiting the activity of degrading enzymes secreted by fungal pathogens [89]. The observed higher accumulation of flavonoids in the resistant HOM+ genotype is in line with the metabolomic studies carried out by Gunnaiah et al. [26] and Dhokane et al. [27], in which flavonoid conjugates with a glucoside or methoxy group were reported to be more abundant in rachis tissues of genotypes carrying the *Fhb1* and *Fhb2* resistance QTL, respectively. Overall, despite the relatively small number of differentially accumulated metabolites at 2 dpi and the nonspecific role of peptides and flavonoids in stress perception and defence metabolisms [23], the present results are indicative of an early onset of plant antioxidant and protective response in the rachis of the resistant HOM+ genotype.

The defence response of the same resistant plants induced at 4 dpi was characterised by a significantly more complex interplay between finely tuned and correlated metabolic pathways (Figure 12), clearly highlighting that several metabolic routes are associated with the FHB resistance conferred by the *Fhb7E* locus. This is in agreement with reports on rachis metabolic and transcriptomic analyses of other loci conferring FHB resistance to durum and bread wheat, including *Fhb1* and *Fhb2* of the Sumai 3 derivation [26,27,51], genes/QTL from Canadian bread and durum wheat germplasm [24,53] and the same *Th. elongatum Fhb7E* locus considered in the present work [69,70]. In line with the common indication from all these studies that rachis and rachis nodes are key sites for the most evident structural and metabolic modifications following *Fg* infection, our durum wheat-*Thinopyrum* spp.-resistant recombinant (*Fhb7E*+) showed upregulation of pathways associated with cell wall adaptation, amino acid and sugar metabolism and flavonoid biosynthesis. Among the most significant features, there was a remarkably increased accumulation of carboxylic acids, such as shikimate and the final product of the shikimate pathway, chorismate (Table S3b), often associated with FHB resistance in wheat (e.g., [23,53]). The shikimate pathway provides substrates for the synthesis of downstream aromatic secondary defence metabolites, such as phenylpropanoids, amines or lignins, while chorismate is a precursor of vitamins K_1_ and B_9_ and of the plant defence hormone salicylic acid [90], and it was previously found to improve resistance to *Fg* in wheat [86,91] and barley [92].

Lignins, phenolic biopolymers generated by the radical coupling of monolignols (hydroxycinnamyl alcohols), are among the most consistently detected induced metabolites associated with FHB resistance in wheat. Although lignin biosynthesis and deposition at the infection site is part of a typical host reaction, regardless of susceptibility [93], their specific role in FHB resistance is consistently associated with main resistance QTL, such as *Fhb1* (e.g., [28,51,94]) and *Fhb2* [27]. In the present study as well, we could confirm that products from the phenylpropanoid pathway (Figure 6), leading to the synthesis of all three types of lignins, accumulated significantly more in the infected resistant HOM+ than in the susceptible HOM− genotype. The association between an increase in lignin abundance, or of its smaller monolignol precursors, and the presence of the *Fhb7E* locus was also observed in the CS-7EL addition line [69,70]. Monolignols from the phenylpropanoid pathway, mainly consisting of coniferyl, p-coumaryl and sinapyl alcohols, give origin to p-hydroxyphenyl (H), guaiacyl (G) and syringyl (S) units, and then are incorporated into lignin polymers [95]. All three lignin types were significantly more accumulated in rachises of the resistant HOM+ vs. HOM− plants (Figure 6). By contrast, only S lignin was more abundant in grains of the HOM+ genotype (Figure 10), possibly because of specific roles of H and G units in lignification and plant defence at the infection site, i.e., floret and rachis in the present context. Since upon pathogen infection lignin H is involved in cell wall growth [96] and lignification [97] more than other units, and low-molecular-weight monomers of H and G units together show some antioxidant capacity [96], this could explain their higher accumulation in rachis. Likewise, the S/G ratio is also associated with changes in the sequestration of molecules involved in defence signals in cell walls [98]. 

An additional interesting feature was represented by detection of a fungal marker metabolite, namely, chitobiose (FC = 2.69, “Carbohydrates and derivatives” class), in rachises of HOM+ plants only. This is indicative of chitinase activity, an enzyme that releases chitobiose when hydrolysing chitin, a structural component of fungal cell walls. Chitinases play a major role as defence proteins in enhancing FHB resistance in wheat [23,99] and are found to be upregulated in FHB-resistant bread wheat “Ning7840” [100] and in the CS-7EL addition line as a 7EL-specific transcript [65].

#### 4.2.2. *Fg*-Induced Metabolic Changes in Grains

Among the major alterations differentiating the HOM+ vs. HOM− grain metabolomes, our study clearly marked the potential contribution of phenolic compounds, such as cinnamic acid and hydroxylated derivatives (including ferulic and sinapic acids), in *Fhb7E*-mediated defence to *Fg* infection, as their concentrations were significantly increased in grains of the resistant vs. the susceptible recombinant (Figure 9 and Figure 10). The accumulation of phenolic compounds in cereal grains is considered a major contributor to the overall antioxidant capacity [101,102]. Moreover, in bread wheat, ferulic acid was found to account for the highest level of hydroxycinnamic in grains [103] and to significantly inhibit *F. graminearum* and *F. culmorum* growth [104,105]. The grain metabolome of the resistant HOM+ plants also showed a tissue-specific upregulation of metabolites of methionine metabolism (Figure 10), which may have contributed to the activation of the phenylpropanoid pathway via ethylene biosynthesis. Ethylene is known to be involved in the response to multiple biotic and abiotic stresses (e.g., [106,107]) and to enhance the initial phenylalanine conversion into cinnamic acid by phenylalanine ammonia-lyase [108]. This reasoning is sustained by the observed higher abundance in the HOM+ genotype of S-adenosylmethionine (SAM), a common precursor of both ethylene and spermidine biosynthesis (Figure 10). As the polyamine content in grains was significantly lower in HOM+ vs. HOM– lines (Figure 10 and ahead), it is reasonable to hypothesise that the SAM intermediate is not utilised for enhancing spermidine synthesis but is rather diverted to other metabolic routes. Opposite directions in accumulation of ethylene and polyamines (spermidine), with higher ethylene content being associated with pathogen resistance, were observed in tomato upon *Botritis cinerea* infection [109] and in peach upon *Monilinia* spp. infection [110]. Our results indicate that also in the case of *Fhb7E* resistance to *Fg*, an ethylene-mediated defence response takes place, which will need further investigation.

### 4.3. Novel Insights into the Metabolomics of FHB Resistance Determined by the Fhb7E Locus

In addition to the metabolites and routes already known to be associated with FHB resistance in wheat, our untargeted metabolomic analysis at 4 dpi was useful for revealing the importance of at least three other, more specific metabolic pathways that significantly differed between the resistant carrier and the susceptible non-carrier of the *Fhb7E* locus. They all involve direct or indirect interactions with the peptide glutathione and consist of polyamine metabolism, VB6 salvage pathway and multiple DON detoxification strategies.

#### 4.3.1. Polyamine Biosynthesis and Conjugation with Glutathione

The specific involvement of polyamines (PAs) in plant responses to pathogen infection is debated, as both plants and pathogens produce PAs, and different mechanisms, e.g., for tissue colonisation and PA transport by the plant, are described for different plant–pathogen interactions [111,112,113]. The picture is complicated by the observed multiple roles of PAs, which represent a source of pathogen growth and virulence but also act as antimicrobial and cell wall protective or signalling molecules, able to activate plant defence upon biotic stress [85,87,114]. Our results showed a clear-cut difference between FHB resistant and susceptible plants in terms of lower accumulation of PA biosynthesis intermediates (“Arginine biosynthesis”) in the former type, in both rachis (Figure 5 and Figure 7) and grain (Figure 8 and Figure 10). Whereas infection by biotrophic/hemi-biotrophic fungi such as *Blumeria graminis, Puccinia hordei* and *Fg*, was reported to induce higher PA accumulation in the resistant vs. susceptible genotypes of wheat, barley and oat [23,82,111], several intermediates of the plant PA biosynthetic pathway (e.g., arginine, citrulline and putrescine) are considered potent inducers of DON production by *Fg* (e.g., [82,87]). The latter function appears to be in line with our observations, as the lower PA abundance in the *Fhb7E*-resistant genotype would constitute an impediment to *Fg* virulence and spread and to DON production [115]. Specifically, putrescine (Put) and spermidine (Spd) were found here to be significantly less abundant in HOM+ vs. HOM– tissues upon *Fg* inoculation (Figure 7 and Figure 10). While Put seems to be mainly involved in modulating *Fg* virulence and DON production in the initial infection stage, Spd accumulates more slowly over the 7 dpi time lapse [82] and may have roles in maintenance of the cell redox homeostasis. Spd is also critical for fungal growth [113,116], DON biosynthesis, environmental stress response and virulence [117]. Hence, by decreasing the availability of Spd in rachis, the resistant HOM+ plants would respond to *Fg* in two ways: by hampering Spd transport to fungus, hyphal growth and virulence, and also by reducing Spd catabolism, which normally results in free radical (H_2_O_2_) release (e.g., [85]). In grains, the accumulation of all intermediates of “Arginine metabolism” (Figure 10) remained low in the resistant HOM+, suggesting a crucial PA protective role at the *Fg* infection site and not in the latter grain development. 

Our results showed that during polyamine biosynthesis, spermidine (Spd), but not spermine (Spm), conjugated with glutathione to form glutathionyl-spermidine (GSpd) in resistant HOM+ plants (Figure 7). GSpd, first discovered in *E. coli* in the mid-1970s, is considered a superior reducing agent to GSH, thus representing a potent free radical scavenger under oxidative stress (reviewed in [118]). Biosynthesis of GSpd is catalysed by a bi-functional GSpd synthetase/amidase (Gss); however, to date, only Enterobacteria and some distantly related eukaryotic Kinetoplastida, most of which are parasites, and a few fungi demonstrate high sequence homology for this enzyme [118,119]. To our knowledge, GSpd has never been identified in plants. The observed accumulation of several antioxidant molecules in the present study is indicative of an oxidative burst occurring during rachis infection, a condition favouring putative GSpd activity in protecting protein and DNA from over-oxidation [118,119]. Moreover, GSH conjugation to the constitutively little available Spd and Spm in HOM+ plants would further limit the catabolism of these PAs, as well as a possible Spm retro-conversion to the critically needed Spd by *Fg* for its growth and virulence [114,116,117]. Nevertheless, there is insufficient evidence here to confirm the existence of a putative GSpd synthetase that can be associated with the GSpd accumulation in the resistant plants. Bearing in mind the remarkable detoxification properties of GSTs towards several xenobiotics, and that knowledge of GST metabolic substrates is still far from being complete [120], the hypothesis of a GSH conjugation to Spd by some GST isoforms (including those of the GTE class encoded by fungal genes, such as that at the *Fhb7E* locus (see [38] and ahead in Concluding remarks), could also be taken into account. A similar metabolism to that of Spd was observed for a higher-order polyamine, aminopropyl-cadaverine (Cad), also accumulated in higher amounts in the resistant plants in the form of glutathionyl conjugate (Figure 7). The role of the Cad and its derivatives in determining plant sensitivity to stress is unclear [121], with some reports hypothesising a Cad role as a ROS-modulating compound [122]. This is also in line with our results, since Cad, as other PAs, is normally catabolised by amino-oxidases to H_2_O_2_, and here it was likely subtracted from oxidation via glutathione conjugation. This suggests possible Cad involvement in the regulation of cell redox homeostasis under *Fg* infection and its significant crosstalk with other PAs.

#### 4.3.2. VB6 Metabolism

VB6 vitamers (pyridoxine, pirydoxal (PL), pirydoxamine and their 5′-phosphorilated derivatives piridoxal 5′-phosphate (PLP), pyridoxine 5′-phosphate (PNP), pyridoxamine 5′-phosphate) are known to have universal and specific roles in plant development, stress tolerance, antioxidant response and amino acid biosynthesis, and also to affect amino acid-dependent secondary metabolites (e.g., [123,124,125,126]. The active form of VB6, PLP, is the best known vitamer that functions as a cofactor for 191 known reactions [127] and can be de novo synthesised or made available by the salvage pathway, i.e., by conversion and phosphorylation of different vitamers into the active PLP form, the latter being the prevailing way [124]. Our results showed significantly higher PLP content in the resistant HOM+ genotype vs. HOM− (Figure 8), together with PNP and PL, indicating a key role of the VB6 salvage pathway in *Fhb7E*-based wheat defence against FHB. In fact, there was no evidence of alteration of the VB6 biosynthesis pathway, as pathways of “Glycolysis” and “Pentose phosphate”, providers of VB6 biosynthesis precursors, were not significantly different in the HOM+ vs. HOM− comparison (Figure 5). The upregulation of genes of the VB6 salvage pathway was also observed in necrotic tissue of potato under *Rhizoctonia solani* infection [128], which agrees with our results. However, some inconsistent results from the expression of salvage pathway genes in tomato under pathogen infection [76] suggest the situation may vary between species, and further analysis is needed. In all cases, to our knowledge this is the first report of differential metabolic profiles for the VB6 complex induced by a biotic stressor (*Fg*) in wheat. The upregulation of VB6 biosynthesis and metabolism was observed in plant adaptation to abiotic stress [123,129], while its role in biotic stress response is rather poorly understood [76], especially in monocot species [125]. Evidence of VB6 complex changes under *Fusarium* spp. infection is reported in a few cases, including *Lilium pumilum* [130], *Vanilla planifolia* [131] and barley [132]. The importance of VB6 in response to plant pathogens is most likely due to its strong antioxidant and ROS scavenging properties [123,127], exerting a similar activity to that of catalase and GST enzymes [128]. In potato necrotic tissue following infection by *Rhizoctonia solani,* three genes from the VB6 pathway and *GST* were upregulated and suppressed pathogen spread [128]. The upregulation of both VB6 and GST was similarly detected in our durum wheat HOM+ line. The potential of VB6 for improving cellular antioxidant capacity is, however, still awaiting more consistent evidence, and the present observations could stimulate further research on wheat and other important crops.

#### 4.3.3. Multiple DON Detoxification Mechanisms

Our investigation on DON accumulation and its fate as bio-transformation products, such as D3G and DON–GSH, in *Fg*-infected rachis tissues of *Fhb7E+* resistant and *Fhb7E*− susceptible lines clearly showed DON content to be remarkably lower in HOM+ samples as compared with HOM− controls after 2 and 4 dpi, respectively (Figure 11a,b). Considering that DON acts as a virulence factor in wheat and that the fungus bursts its production at the rachis node [133,134], this observation is per se relevant as a limiting condition to fungal spread. Nevertheless, we observed that whereas DON was the only metabolite detected at 2 dpi in both HOM+ and HOM− plants, after 4 dpi, D3G and DON–GSH were differentially present in susceptible and resistant lines. The D3G metabolite, derived from UGT-based glycosylation, was detected in infected rachis tissues of both susceptible and resistant plants, although its relative abundance was significantly higher in resistant samples (Figure 11). This observation suggests that this mechanism of DON detoxification is present and active in both genotypes but is more efficient or more rapidly activated in the HOM*+* resistant line. Concerning the DON–GSH metabolite, it was exclusively detected in resistant samples. Thus, DON to glutathione conjugation is likely to be a key and distinctive mechanism of DON detoxification determined by the presence of the *Fhb7E* locus. Interestingly, the further involvement of GSH in detoxification mechanisms was highlighted by a recent study in tomato, where GSH was found to positively regulate the expression of UGT genes in response to a pesticide-induced stress [135]. If the same crosstalk mechanism among seemingly independent plant protective paths would also be active in our system, it would explain the enhancement of both DON–GSH and D3G observed in *Fhb7E* carriers compared with the control line. Whatever the mechanism, the reduction of total DON content in *Fhb7E*+ wheat grains (Figure 11) is particularly important for food and feed safety [136].

## 5. Concluding Remarks

*Fhb7E* transfer has garnered much interest, primarily for its remarkable effect towards *Fusarium* diseases and consequent breeding value, but also for its rather unusual biological implications. Not only it is an alien transfer from a wild wheat relative, but also, in turn, it incorporates a critical gene derived from a sexually unrelated, endophytic fungal species (*Epichloë* genus). In the original evidence provided by Wang et al. [38], the HGT event was assumed to be restricted to Triticeae host species belonging to the *Thinopyrum* genus. More recently, *Fhb7* homologs were found in additional genera of the Triticeae tribe, such as *Elymus*, *Leymus*, *Roegneria* and *Pseudoroegneria*, which led to the hypothesised occurrence of the HGT event before Triticeae differentiation [63,64]. Notably, all species reported as *Fhb7* carriers exhibit a perennial habit, as in other gene transfer episodes from *Epichloë* into grass species [137,138]. Although several aspects of the HGT phenomenon in Eukaryotes remain to be further investigated [139,140], it is tempting to speculate that perenniality, an ancestral condition of angiosperms vs. annuality [141], might have facilitated the establishment and long-term maintenance of the endophyte–plant interaction, thereby increasing the adaptive and functional advantages of both the fungal and the host plant partners. If so, this might be one of the reasons to explain why the *Fhb7* transfer did not apparently involve typically annual *Triticum* species. Nonetheless, the *Epichloë*-to-*Thinopyrum* spp. HGT was made available and could benefit cultivated *Triticum* species thanks to finely tailored chromosome engineering interventions, which apparently did not cause negative impacts on yield performance of the recipient wheat genotypes (e.g., [62,63,66,71]).

Our data clearly show the importance of the *Fhb7E*-associated resistance of glutathione (GSH), a potent ROS scavenger and a key molecule in DON detoxification, both as a substrate for GST activity and, possibly, as a positive regulator of UGTs. In this regard, it could be hypothesised that the HGT-acquired GST, belonging to the fungal GTE (glutathione transferase etherase-related) class of GST proteins [38], not found in wheat and in plants in general [36], could integrate the GST arsenal of the host plant, conferring even more effective/diversified abilities to these multi-functional proteins. Besides for GSH and genes/compounds related to its metabolism, we have added new evidence of the importance of polyamines and vitamin B_6_ in *Fhb7E*-mediated resistance. It is reasonable to think that the wide array of metabolites and pathways differentiating the *Fhb7E*+ from the *Fhb7E*− response can hardly be attributed to the alien *GST* gene alone. Not disregarding the importance of interactions of the *Thinopyrum* locus with the background wheat genotype, additional *cis*- or *trans*-active genes, with structural and regulatory roles, might be expected to be involved in the makeup of the critical *Thinopyrum* region and hence to contribute to the *Fhb7E*-associated phenotype. In this view, it can be supposed that only when the fungal *GST* sequence “landed” into a “suitable genetic environment”, such as that of distal *Thinopyrum* 7EL arm portion, flanked by additional genes involved in the resistance function (see Introduction), did the composite locus become able to confer the observed outstanding resistance to FHB and also to Fusarium crown rot [62,66]. Such flanking sequences might be absent or have less/no active alleles in those transgenic and wheat *Thinopyrum* lines, which failed to show FHB resistance, despite having been proven to possess a highly homologous or an identical *GST* gene as the resistant genotypes [63,64,142]. The hypothesised compound nature of the *Fhb7E* locus matches the recent description of numerous clusters in the bread wheat genome of *Fg*-responsive genes, containing a variety of functionally related and largely co-expressed defence genes [143]. Many of such gene clusters turned out to be linked to, or in the vicinity of, known FHB QTL, and the present research provides novel insights for a comprehensive view of one of the most effective of such QTL for arming wheat with an effective defence against *Fusarium* diseases.

## Data Availability

The datasets used and/or analysed during the current study are available in Supplementary Material here and from the corresponding authors upon reasonable request.

## Supplementary Materials

The following supporting information can be downloaded at: https://www.mdpi.com/article/10.3390/cells12081113/s1, Table S1: Segregation and χ^2^ test (1:2:1 ratio of HOM+:HET:HOM− genotypes) of BC_3_F_2_ progenies of R69-9/R5 recombinant types; Table S2: List of resistance-related constitutive, RRC (**a**) and induced, RRI (**b**) metabolites significantly more accumulated in rachises of HOM+ vs. HOM− genotype at 2 days post-inoculation; Table S3: List of resistance-related constitutive, RRC (**a**) and induced, RRI (**b**) metabolites significantly more accumulated in rachises of HOM+ vs. HOM− genotype at 4 days post-inoculation; Figure S1: Metabolomics Pathway Analysis (MetPA) of rachis tissue from the resistant *Fhb7E* carrier (HOM+) and susceptible non-carrier (HOM−) lines at 4 dpi after water (*mock*) or *Fg* treatment: (a) *Fg*-HOM+ vs. *mock*-HOM+, and (b) *Fg*-HOM– vs. *mock*-HOM− comparisons; Figure S2: (A) EICs of DON–GSH catalysed by the *Fhb7E-GST* gene in *Fg*-inoculated *Fhb7E*+ (green) and *Fhb7E*− rachis samples (yellow). (B) Base peak chromatogram of DON–GSH precursor ion (*m*/*z* 604.2173, retention time 7.03 min) of *Fg*-inoculated *Fhb7E*+ samples. (C) Tandem mass spectra generated by fragmentation of the DON–GSH precursor ion.
