# Supplementary material for: Untargeted Metabolomics Reveals a Multi-Faceted Resistance Response to Fusarium Head Blight Mediated by the Thinopyrum elongatum Fhb7E Locus Transferred via Chromosome Engineering into Wheat
Source: Cells. 2023 Apr 8;12(8):1113. doi: 10.3390/cells12081113 (PMC10136595; doi:10.3390/cells12081113)

Supplementary material

Untargeted metabolomics reveals a multi-faceted response to Fusarium Head Blight mediated by the *Thinopyrum elongatum Fhb7E* locus transferred via chromosome engineering into wheat

Giuseppina Fanelli, Ljiljana Kuzmanović, Gloria Giovenali, Silvio Tundo, Giulia Mandalà, Sara Rinalducci and Carla Ceoloni

Table S1. Segregation and χ^2 test (1:2:1 ratio of HOM+ : HET : HOM- genotypes) of BC_3F_2 progenies of R69-9/R5 recombinant types (HOM+, and HET, plants homozygous and heterozygous, respectively, for the alien introgression; HOM-, plants lacking the alien introgression; *p*, probability; ns, non-significant).

Progeny	Seed No.	HOM+	HET	HOM-	χ^2	*p*
V18x65-1	123	35	65	23	2.74	0.254 ^{ns}
V18x65-3	51	14	26	11	0.373	0.830 ^{ns}
V18x70-1	29	8	16	5	0.931	0.628 ^{ns}
V18x70-2	9	1	7	1	2.778	0.249 ^{ns}
V18x72-1	20	4	9	7	1.1	0.577 ^{ns}
Total	232	62	123	47	2.784	0.249 ^{ns}

Table S2. List of resistance-related constitutive, RRC (**a**) and resistance-related induced, RRI (**b**) metabolites significantly more accumulated in rachises of HOM+ vs. HOM- genotype at 2 days post inoculation (FC > 1.5; FC = fold change).

	No.	Metabolite	Chemical classification	FC
**a)**	1	Agmatine	Amino acid and amino acid derivative	96.381
	2	Gibberellin A24	Terpenoids	4.182
	3	Adifoline	Alkaloids	3.532
	4	2S-Amino-tridecanoic acid	Lipid	3.363
	5	5--Phosphoribosyl-N-formylglycinamide	Carbohydrate and derivates	2.777
	6	Jamaicamide B	Lipid	2.660
	7	Glabric acid	Terpenoids	2.627
	8	2-6-Diamino-7-hydroxy-azelaic acid	Amino acid and amino acid derivative	2.612
	9	10-Deoxysarpagine	Other	2.580
	10	DIBOA-glucoside	Carbohydrate and derivates	2.578
	11	Cimigenol	Terpenoids	2.512
	12	S-(Hydroxyphenylacetothiohydroximoyl)-L-cysteine	Other	2.451
	13	Taxifolin 3-O-acetate	Flavonoids	2.344
	14	N6-N6-Dimethyladenosine	Carbohydrate and derivates	2.320
	15	cyclo-Dopa 5-O-glucoside	Phenols	2.297
	16	L-Citrulline	Amino acid and amino acid derivative	2.258
	17	Flavonol 3-O-beta-D-glucosyl-(1->2)-beta-D-glucoside	Flavonoids	2.199

18	Eupachloroxin	Terpenoids	2.195
19	dCMP	Nucleotides	2.127
20	Peptidylglycine	Other	2.086
21	Heliotrine	Alkaloids	2.009
22	N-Feruloylglycine	Carboxylic Acids	1.992
23	Vitexin 2---O-beta-D-glucoside	Flavonoids	1.977
24	Lonchocarpenin	Flavonoids	1.918
25	Fruticosonine	Indoles	1.866
26	Fagaramide	Carboxylic Acids	1.866
27	Aromaticin	Terpenoids	1.863
28	N-(3-Oxohexanoyl)homoserine lactone	Lipid	1.821
29	17-Hydroxylinolenic acid	Lipid	1.801
30	N-Acetylneuraminate 9-phosphate	Other	1.798
31	Gradolide	Terpenoids	1.793
32	Deacetylvindoline	Alkaloids	1.778
33	Stearidonic acid	Lipid	1.777
34	Bruceine B	Terpenoids	1.767
35	Pyridine-2-3-dicarboxylate	Heterocyclic Compounds	1.766
36	O-Acetylcypholophine	Other	1.714
37	Linalyl acetate	Terpenoids	1.683
38	Indolelactate	Indoles	1.660
39	Auriculine	Carbohydrate and derivates	1.652
40	Paeonoside	Carbohydrate and derivates	1.635
41	(9Z-12Z-15Z)-(7S-8S)-Dihydroxyoctadeca-9-12-15-trienoic acid	Lipid	1.621
42	6-(alpha-D-Glucosaminyl)-1D-myo-inositol	Carbohydrate and derivates	1.620
43	Astrocasine	Other	1.619
44	Xanthyletin	Heterocyclic Compounds	1.616
45	Spermine	Amines	1.614
46	(S)-2-Hydroxystearate	Lipid	1.606
47	sn-glycero-3-Phosphocholine	Lipid	1.595
48	(-)-Phaseollin	Flavonoids	1.591
49	Abyssinone I	Flavonoids	1.591
50	DIBOA	Heterocyclic Compounds	1.587
51	2-Methyl-3-hydroxy-5-formylpyridine-4-carboxylate	Other	1.587
52	Falaconitine	Alkaloids	1.584
53	Salicin 6-phosphate	Glycosides	1.584
54	Porphobilinogen	Other	1.565
55	Oleoylglycerone phosphate	Carbohydrate and derivates	1.559
56	2-C-Methyl-D-erythritol 4-phosphate	Carbohydrate and derivates	1.548
57	N-Methylethanolamine phosphate	Other	1.546
58	Amylopectin	Polymers	1.546
59	N2-Succinyl-L-arginine	Amino acid and amino acid derivative	1.527
60	Asclepin	Carbohydrate and derivates	1.506
61	Glycyphyllin	Carbohydrate and derivates	1.504

b)

1	Flavonol 3-O-D-xylosylglycoside	Flavonoids	3.085
2	Heliamine	Alkaloids	2.807
3	Pyridoxine phosphate	Other	2.593
4	5-Hydroxymethyldeoxycytidylate	Carbohydrate and derivates	2.588
5	Leucyl-leucine	Peptides	2.366
6	Glutathione	Peptides	2.066
7	5-Hydroxyindoleacetylglycine	Other	1.970
8	Abscisic acid glucose ester	Carboxylic Acids	1.957
9	2-N-6-N-Bis(2-3-dihydroxy-N-benzoyl)-L-serine	Other	1.750
10	1-(5-Phosphoribosyl)-4-(N-succinocarboxamide)-5-aminoimidazole	Other	1.713
11	1-Deoxy-D-altro-heptulose 7-phosphate	Carbohydrate and derivates	1.694

12	gamma-L-Glutamylputrescine	Amino acid and amino acid derivative	1.691
13	Actinamine	Other	1.599
14	N-Acetyl-beta-D-glucosaminyl-1-3-N-acetyl-D-galactosaminyl-R	Carbohydrate and derivates	1.564
15	GMP	Nucleotides	1.530
16	Apigenin 7-4--dimethyl ether	Flavonoids	1.526

Table S3. List of resistance-related constitutive, RRC (**a**) and resistance-related induced, RRI (**b**) metabolites significantly more accumulated in rachises of HOM+ vs. HOM- genotype at 4 days post inoculation (FC > 1.5; FC = fold change).

	No.	Metabolite	Chemical classification	FC
**a)**	1	Agmatine	Amino acid related compounds	57.756
	2	N6-Alkylaminopurine-9-beta-D-glucoside	Others	3.416
	3	N-Hydroxyl-tryptamine	Indoles	3.254
	4	Acetylagmatine	Guanidines	2.992
	5	Abscisic acid glucose ester	Carboxylic Acids	2.932
	6	Deoxyuridine	Nucleosides	2.676
	7	N-Heptanoylhomoserine lactone	Carboxylic acid	2.571
	8	3--4--5-6-Tetrahydroxy-3-7-dimethoxyflavone	Flavonoids	2.247
	9	D-glucosamine-6-phosphate	Carbohydrates	2.042
	10	S-Decyl GSH	Peptides	2.041
	11	Glutathionylspermidine	Amines	2.000
	12	5--Phosphoribosylglycinamide	Secondary metabolites	1.972
	13	xanthine	Alkaloids	1.911
	14	Flavonol 3-O-beta-D-glucosyl-(1->2)-beta-D-glucoside	Flavonoids	1.833
	15	5-Hydroxyindoleacetylglycine	Others	1.825
	16	Hydroxyvernolide	Terpenoids	1.791
	17	9-Methylthiononanaldoxime	Others	1.766
	18	1-Guanidino-1-deoxy-scyllo-inositol 4-phosphate	Organic hydroxy compound	1.751
	19	leucine/isoleucine	Amino acid and amino acid derivative	1.723
	20	Flindersiamine	Alkaloids	1.701
	21	Hexahomomethionine	Amino acid and amino acid derivative	1.659
	22	Erysonine	Alkaloids	1.625
	23	N-Nitrosodiphenylamine	Others	1.624
	24	Fastigilin C	Terpenoids	1.610
	25	Deoxyinosine	Nucleosides	1.598
	26	N-Dimethyl-2-aminoethylphosphonate	Others	1.598
	27	Robinobiose	Carbohydrates	1.591
	28	Gibberellin A24	Terpenoids	1.564
	29	Indoleamine	Indoles	1.556

**b)**	1	N1-(5-Phospho-alpha-D-ribosyl)-5-6-dimethylbenzimidazole	Carbohydrate and derivates	112.21
	2	shikimate	Carboxylic acid	58.451
	3	L-2-Methyltryptophan	Amino acid and amino acid derivative	51.389
	4	N-Caffeoylputrescine	Phenylpropanoid	35.944
	5	6-Oxochelerythrine	Heteromonocyclic compound	12.940
	6	7-Methylxanthosine	Alkaloids	11.076
	7	Protein N6-methyl-L-lysine	Amino acid and amino acid derivative	10.388
	8	Leucyl-leucyl-norleucine	Peptides	8.780
	9	5-o-Feruloyquinic acid	Phenylpropanoid	7.406
	10	S-Hexyl-glutathione	Peptides	7.255
	11	1-Oleoylglycerophosphocholine	Lipid	7.255

12	Acremoauxin A	Indoles	6.910
13	D-Glucosaminide	Other	6.652
14	2-Carboxy-D-arabinitol	Carbohydrate and derivates	6.434
15	4-Prenylresveratrol	Phenylpropanoid	6.375
16	Acetylblasticidin S	Heteromonocyclic compound	6.231
17	Caffeoquinone	Carboxylic acid	6.228
18	Oxaloglutarate	Carboxylic acid	6.158
19	D-Glucono-1-5-lactone 6-phosphate	Other	6.016
20	2-Demethylmenaquinone	Other	5.845
21	N-Feruloylglycine	Carboxylic acid	5.795
22	N2-(D-1-Carboxyethyl)-L-lysine	Amino acid and amino acid derivative	5.790
23	1-Octen-3-ol-3-o-beta-D-xylopyranosyl(1->6)-beta-D-glucopyranoside	Carbohydrate and derivates	5.763
24	N(6)-[(Indol-3-yl)acetyl]-L-lysine	Heterocyclic Compounds	5.507
25	N-Formyl-D-kynurenine	Amino acid and amino acid derivative	5.492
26	N-Acyl-L-arginine	Amino acid and amino acid derivative	5.226
27	S-(Hydroxymethyl)glutathione	Peptides	5.146
28	N-Acetyl-L-glutamate 5-phosphate	Amino acid and amino acid derivative	5.090
29	Prephenate	Carboxylic acid	4.828
30	Cellotetraose	Carbohydrate and derivates	4.550
31	Brusatol	Terpenoids	4.437
32	6-Acetyl-beta-D-galactoside	Carbohydrate and derivates	4.401
33	2-(5--Methylthio)pentylmalic acid	Carboxylic acid	4.391
34	Callichiline	Heterocyclic Compounds	4.230
35	methylquercetin	Flavonoids	4.215
36	Gibberellin A12 aldehyde	Terpenoids	4.183
37	Gallate	Phenols	3.997
38	2-Hydroxyethylphosphonate	Other	3.996
39	Gibberellin A5	Terpenoids	3.989
40	Glutathione	Peptides	3.960
41	5-Methylcytosine	Heterocyclic Compounds	3.936
42	Geranyl diphosphate	Terpenoids	3.756
43	Cysteinyldopa	Amines	3.695
44	Ammoresinol	Terpenoids	3.598
45	DIBOA	Heterocyclic Compounds	3.497
46	Caribine	Alkaloids	3.490
47	NAC	Carboxylic acid	3.418
48	N-Acetyl-4-O-acetylneuraminate	Carboxylic acid	3.417
49	Isochamaejasmin	Flavonoids	3.351
50	Paeonoside	Carbohydrate and derivates	3.243
51	Pipecolic acid	Carboxylic acid	3.145
52	Sinapyl alcohol	Phenylpropanoid	3.129
53	glucose-6-phosphate	Carbohydrate and derivates	3.114
54	Hydroxymethylphosphonate	Other	3.091
55	Geranyl-hydroxybenzoate	Terpenoids	3.072
56	1-Alkyl-2-acylglycerophosphoethanolamine	Other	2.988
57	Thiamine aldehyde	Vitamins	2.977
58	2-3-Dehydro-gibberellin A9	Terpenoids	2.956
59	Acetylcysteine	Carboxylic acid	2.929
60	Acronycidine	Alkaloids	2.884
61	Menaquinone	Vitamins	2.862
62	TRIBOA	Heterocyclic Compounds	2.845
63	1-Hydroxyalkyl-sn-glycerol	Other	2.838
64	6-Methoxytaxifolin	Flavonoids	2.835
65	Soraphen O	Other	2.824
66	2-Methylpropanoyl phosphate	Other	2.816
67	8-Epideoxyloganin	Terpenoids	2.804
68	S-Octyl GSH	Peptides	2.780

69	N(alpha)-t-Butoxycarbonyl-L-leucine	Amino acid and amino acid derivative	2.773
70	8-Methylthiooctyl glucosinolate	Other	2.712
71	Chitobiose	Carbohydrate and derivates	2.697
72	N-Acetyldemethylphosphinothricin tripeptide	Peptides	2.620
73	Indolepyruvate	Indoles	2.580
74	CMP-N-glycoloylneuraminate	Other	2.566
75	Altersolanol A	Phenols	2.549
76	5-O-Caffeoylshikimic acid	Phenylpropanoid	2.549
77	Orotate	Carboxylic acid	2.548
78	Quercetin 3-O-(6-O-malonyl-beta-D-glucoside)	Flavonoids	2.450
79	3-Sulfino-L-alanine	Amino acid and amino acid derivative	2.423
80	Citrinin	Mycotoxins	2.421
81	(3S-4S)-3-Hydroxytetradecane-1-3-4-tricarboxylate	Lipid	2.412
82	N-Monomethyl-2-aminoethylphosphonate	Other	2.412
83	Anthocyanidin	Flavonoids	2.402
84	Inosine	Nucleotides and derivates	2.341
85	1-(5-Phosphoribosyl)-4-(N-succinocarboxamide)-5-aminoimidazole	Other	2.328
86	1-Organyl-2-lyso-sn-glycero-3-phosphocholine	Lipid	2.319
87	Glutathione disulfide	Peptides	2.285
88	pyridoxine	Vitamins	2.265
89	N-Acetyl-L-citrulline	Carboxylic acid	2.220
90	3--5--Cyclic IMP	Nucleotides and derivates	2.216
91	1-O-Feruloyl-beta-D-glucose	Phenols	2.211
92	5-Hydroxyconiferaldehyde	Phenylpropanoid	2.193
93	N-(4-Guanidinobutyl)-4-hydroxycinnamide	Phenols	2.185
94	7-Methylthioheptanaldoxime	Other	2.178
95	Fruticosonine	Alkaloids	2.176
96	Feruloylputrescine	Carboxylic acid	2.160
97	S-Methyl-1-thio-D-glycerate	Other	2.156
98	2-6-Dihydroxyanthraquinone	Phenols	2.135
99	Hordatine A	Phenols	2.129
100	Gnididilatin	Terpenoids	2.128
101	5-Hydroxypentanoate	Carboxylic acid	2.101
102	Allantoin	Heterocyclic Compounds	2.088
103	trans-2-3-Dihydroxycinnamate	Carboxylic acid	2.082
104	L-Homocysteic acid	Amino acid and amino acid derivative	2.043
105	N-Acetyllactosamine	Carbohydrate and derivates	2.025
106	Pyridoxal phosphate	Vitamins	2.025
107	Cucurbic acid	Lipid	2.008
108	alpha-ketoglutarate	Carboxylic acid	1.986
109	Adenosine	Nucleotides and derivates	1.981
110	Caffeate	Phenylpropanoid	1.967
111	(R)-Mevalonate	Carboxylic acid	1.917
112	lupinosoflavone G	Flavonoids	1.868
113	D-Erythritol 4-phosphate	Carbohydrate and derivates	1.858
114	10-Deoxygeniposidic acid	Terpenoids	1.850
115	Chorismate	Carboxylic Acids	1.828
116	Caracurine V	Alkaloids	1.819
117	5-Hydroxykynurenine	Amino acid and amino acid derivative	1.760
118	2-Oxoglutaramate	Carboxylic acid	1.760
119	Cinegalline	Alkaloids	1.748
120	N2-Succinyl-L-arginine	Carboxylic acid	1.737
121	N-Acetyl-D-quinovosamine	Carbohydrate and derivates	1.733
122	Phosphorylcholine	Lipid	1.732
123	10-Deoxysarpagine	Other	1.725
124	L-Serine	Amino acid and amino acid derivative	1.710
125	(9R-10R)-Dihydroxyoctadecanoic acid	Lipid	1.705

126	N-Succinyl-LL-2-6-diaminoheptanedioate	Carboxylic acid	1.681
127	6-8-Diprenylnaringenin	Flavonoids	1.671
128	Berbamunine	Alkaloids	1.633
129	Diferulic acid	Phenylpropanoid	1.621
130	Xylose	Carbohydrate and derivates	1.620
131	Pantothenol	Vitamins	1.602
132	2(alpha-D-Mannosyl)-D-glycerate	Carbohydrate and derivates	1.599
133	Flavonol 3-O-D-xylosylglucoside	Flavonoids	1.596
134	Leucyl-leucine	Peptides	1.578
135	Succinylproline	Carboxylic acid	1.567
136	Lipoate	Lipid	1.552
137	L-Histidinol phosphate	Other	1.547
138	(R)-3-((R)-3-Hydroxybutanoyloxy)butanoate	Carboxylic acid	1.532
139	N6-Methyl-L-lysine	Amino acid and amino acid derivative	1.529
140	Pyridoxine phosphate	Vitamins	1.529
141	6--Dehydro-6--oxoparomamine	Other	1.517
142	2--Deoxymugineic acid	Carboxylic acid	1.514
143	N-Adenylyl-L-phenylalanine	Amino acid and amino acid derivative	1.510

Figure S1. Metabolomics Pathway Analysis (MetPA) of rachis tissue from the resistant *Fhb7E* carrier (HOM+) and susceptible non-carrier (HOM-) lines at 4 dpi after water (*mock*) or *Fg* treatment: (**a**) *Fg*-HOM+ vs. *mock*-HOM+, and (**b**) *Fg*-HOM- vs. *mock*-HOM- comparisons. All the matched pathways are displayed as circles. The colour of each circle is based on *p*-values (darker colours indicate more significant changes of metabolites in the corresponding pathway), whereas the size of the circle corresponds to the pathway impact score. The most impacted pathways having high statistical significance scores are annotated by their full name or numbers (see legend).

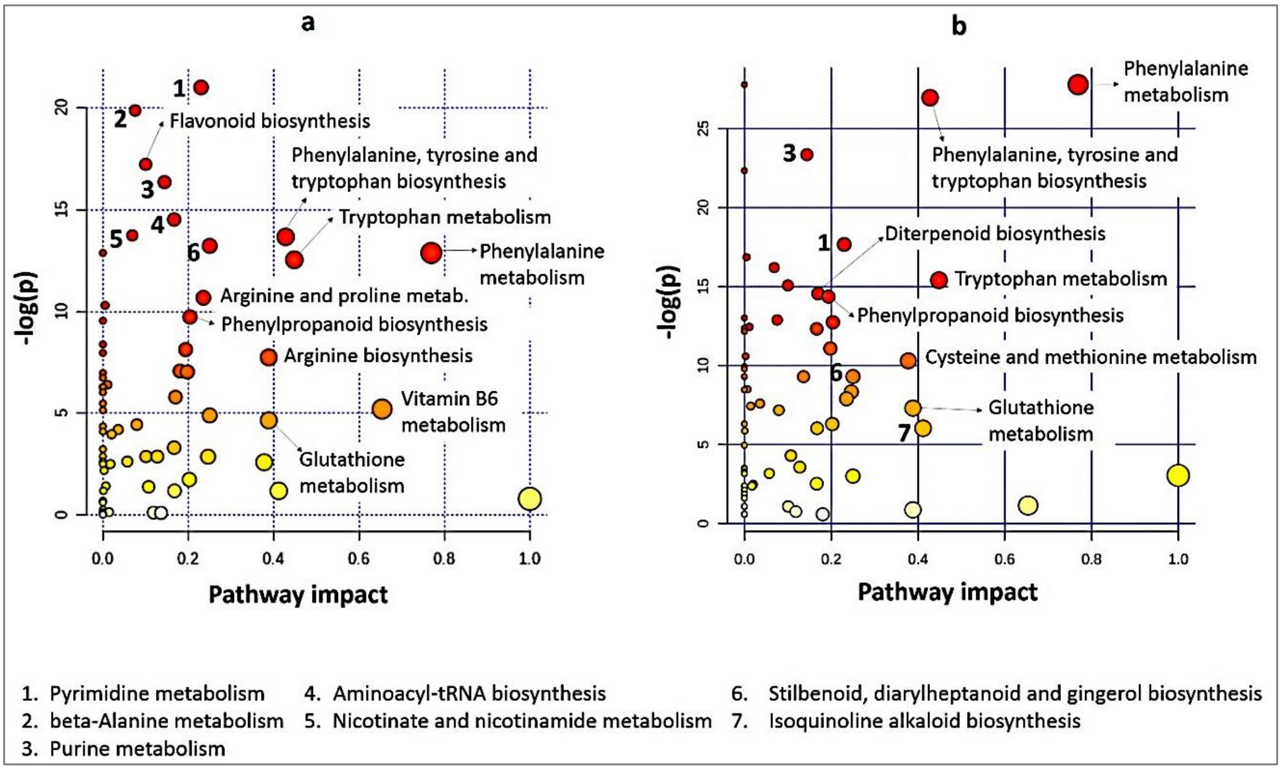

Figure S2. **A**) EICs of DON-GSH catalyzed by the *Fhb7E-GST* gene in *Fg*-inoculated *Fhb7E+* (green) and *Fhb7E-* rachis samples (yellow). **B**) Base peak chromatogram of DON-GSH precursor ion (m/z 604.2173, retention time 7.03 min) of *Fg*-inoculated *Fhb7E+* samples. **C**) Tandem mass spectra generated by fragmentation of the DON-GSH precursor ion. The m/z values for possible fragments and the corresponding formula are indicated.

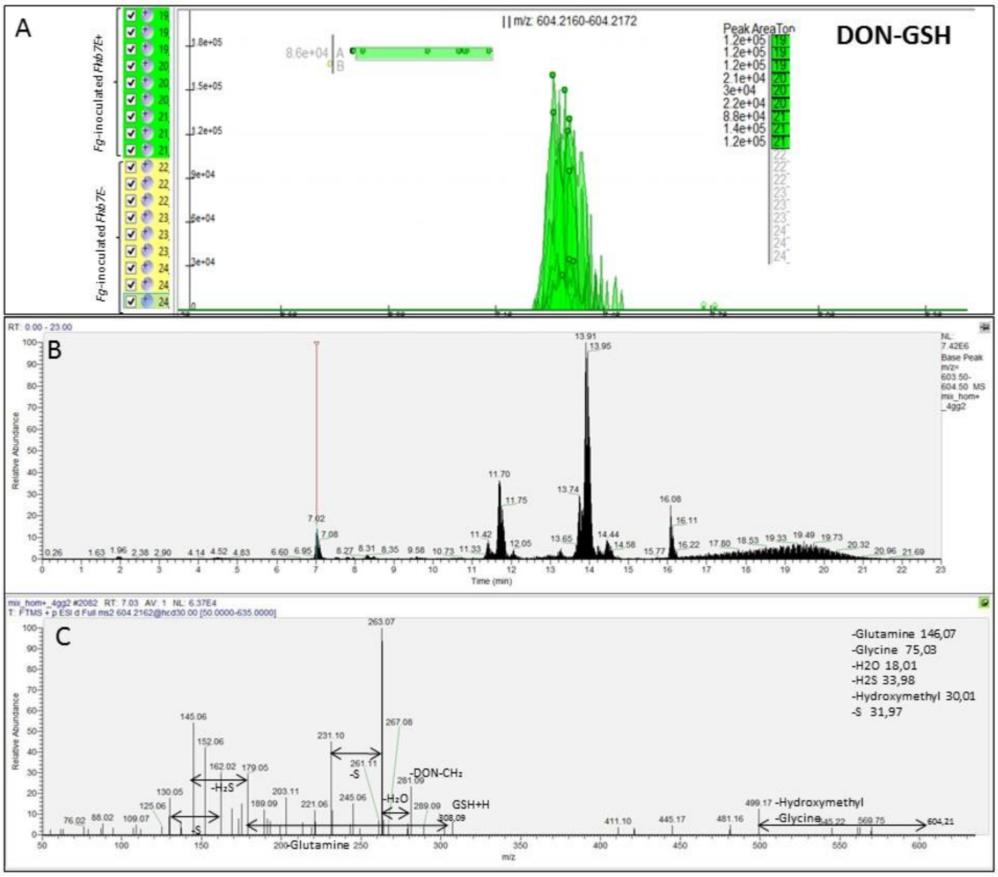

Supplement: Supplementary file 1 [file cells-12-01113-s001.zip › cells-2279791-supplementary.pdf]
